# Comparative treatment planning of photon, proton and carbon ion radiotherapy for sphenoid wing meningiomas

**DOI:** 10.1016/j.phro.2026.101018

**Published:** 2026-06-13

**Authors:** Sophie Rauh, Maximilian Y. Deng, Inga Jessen, Lisa Seidel, Line Hoeltgen, Semi Harrabi, Filipa Baltazar, Thomas Haberer, Andrea Mairani, Klaus Herfarth, Jürgen Debus, Thomas Tessonnier, Laila König

**Affiliations:** a Department of Radiation Oncology, Heidelberg University Hospital, Heidelberg University, Im Neuenheimer Feld 400, 69120 Heidelberg, Germany; b Heidelberg Institute for Radiation Oncology (HIRO) and National Center for Radiation Research in Oncology (NCRO), Im Neuenheimer Feld 400, 69120 Heidelberg, Germany; c National Center for Tumor Diseases (NCT), NCT Heidelberg, a partnership between DKFZ and Heidelberg University Hospital, Im Neuenheimer Feld 460, 69120 Heidelberg, Germany; d Heidelberg Ion-Beam Therapy Center (HIT), Department of Radiation Oncology, Heidelberg University Hospital, Heidelberg University, Im Neuenheimer Feld 450, 69120 Heidelberg, Germany; e Hopp Children's Cancer Center Heidelberg (KiTZ), Im Neuenheimer Feld 430, 69120 Heidelberg, Germany; f Clinical Cooperation Unit Radiation Oncology, German Cancer Research Center (DKFZ), Im Neuenheimer Feld 280, 69120 Heidelberg, Germany; g Medical Faculty, Heidelberg University, Im Neuenheimer Feld 672, 69120 Heidelberg, Germany; h Clinical Cooperation Unit Translational Radiation Oncology, German Cancer Research Center (DKFZ), Im Neuenheimer Feld 280, 69120 Heidelberg, Germany; i Centro Nazionale di Adroterapia Oncologica (CNAO), Medical Physics Department, Via Erminio Borloni, 1, 27100 Pavia, Italy

**Keywords:** Sphenoid wing meningioma, Proton radiotherapy, Carbon ion radiotherapy, Treatment plan comparison, Normal tissue complication probability, Radiation-induced CNS malignancies

## Abstract

**Background and purpose:**

Sphenoid wing meningiomas (SWMs) are located adjacent to critical organs of interest (OOIs) and present challenges for surgery and radiotherapy. Given the favorable overall survival in meningioma patients, preserving quality of life is important. Dose reductions within OOIs may lower adverse events. This study aimed to evaluate potential advantages of particle therapy regarding dose distribution and side effects for SWMs.

**Materials and methods:**

Nine patients (eight females, one male; median age at radiotherapy: 55 years) with SWM received proton radiotherapy (PRT, 54 Gy (RBE), 1.8 Gy (RBE) per fraction). Comparative treatment plans were generated for volumetric modulated arc therapy (VMAT, 54 Gy, 1.8 Gy per fraction) and carbon ion radiotherapy (CIRT, 42 Gy (RBE), 3 Gy (RBE) per fraction). Target volumes and OOI dose guidance were maintained. OOI dose-volume parameters, normal tissue complication probabilities (NTCP), and risk ratios of radiation-induced secondary central nervous system (CNS) malignancies were assessed.

**Results:**

Compared to VMAT, mean relative brain doses were reduced by −41.5% with PRT, and − 63.8% with CIRT. Particle therapy achieved significant dose sparing of bilateral hippocampi and lenses, and the ipsilateral inner ear. NTCPs indicated lower risks of ipsilateral hearing loss and cataract with particle therapy. The mean estimated risk of radiation-induced secondary malignancies was 1.7 for photons over protons, and 2.7 for photons over carbon ions.

**Conclusions:**

For SWMs, particle therapy demonstrated reduced dose exposure to several OOIs, and may lower the risk of side effects compared with VMAT.

## Highlights

• Particle therapy showed superior dose distribution for sphenoid wing meningiomas.
• Dose deposition to bilateral hippocampi and lenses was reduced with particle therapy.
• Model-based calculations indicated a risk reduction for cataract and hearing loss.
• The absolute risk of delayed recall was reduced by −34% with carbon ions vs.
• photons.
• Photons showed higher secondary CNS malignancy risks than protons and carbon ions.

## Graphical abstract

**Figure:**
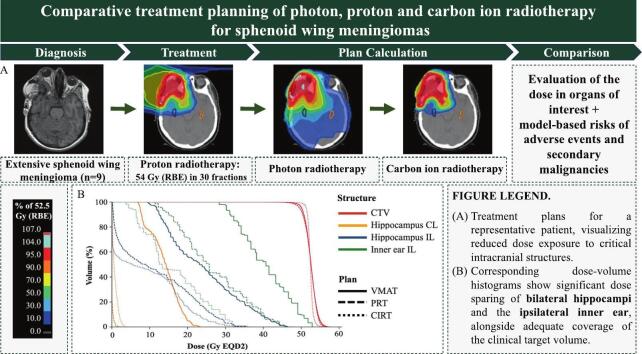

## 1 Introduction

Meningiomas represent the most prevalent brain tumor entity in adults, comprising approximately 41.7% of cases [1]. Over 80% are benign, WHO grade 1 meningiomas, typically associated with favorable prognoses, emphasizing the significance of preserving quality of life [1], [2]. The median age at diagnosis is 68 years, and the female-to-male ratio is 2.3:1 [1]. The EANO guideline recommends surgical resection as first-line treatment for enlarging or symptomatic meningiomas, aiming for a maximum safe resection [3]. However, gross total resection is often not feasible in skull base meningiomas (SBMs) due to anatomical constraints, and radical surgery may result in cranial nerve or neuropsychological deficits [4], [5], [6]. For patients not safely amenable to resection, particularly in SBMs due to their proximity to critical organs of interest (OOIs), definitive radiotherapy is recommended [3].

Definitive photon radiotherapy (XRT) and particle radiotherapy for benign SBMs demonstrated 5-year progression-free survival (PFS) rates frequently exceeding 90–95% [7], [8], [9], [10], [11], [12]. However, patients with sphenoid wing meningiomas (SWMs) may develop radiation-induced adverse events, including visual or auditory impairments, neurocognitive decline, and secondary malignancies [7], [13], [14], [15], [16], [17]. Particle therapy, such as proton radiotherapy (PRT) and carbon ion radiotherapy (CIRT), utilizes the distinct depth-dose profile of charged particles, leading to a sharply increased energy deposition within circumscribed regions at the end of the particle's range, referred to as the Bragg Peak [18], [19]. Given the limited global availability of particle therapy, it is critical to identify indications and OOIs for which particle therapy can achieve substantial dose reductions, in order to prioritize patients most likely to benefit [20].

The present study compared dose-volume parameters of the target volume and critical OOIs between PRT, volumetric modulated arc therapy (VMAT), and CIRT for extensive SWMs in patients previously treated with PRT. Furthermore, it estimated the risk of radiation-induced adverse events in selected OOIs through normal tissue complication probability (NTCP) models, and the risk of radiation-induced secondary central nervous system (CNS) malignancies associated with each modality. The aim was to assess whether particle therapy may reduce dose exposure to critical OOIs and associated side effects in extensive SWMs.

## 2 Materials and methods

### 2.1 Clinical patient characteristics and proton radiotherapy

This study included nine exemplary patients (eight females, one male) with an extensive SWM, confirmed histologically (56%, WHO grade 1) or radiologically by DOTATOC-PET-CT (44%), who received definitive PRT at the Heidelberg Ion-Beam Therapy Center (HIT) between 2014 and 2022, according to standard-of-care protocols. Target volume delineation, dose prescriptions, and treatment planning followed current clinical guidelines, and are illustrated in Supplementary Material A. A total dose of 54 Gy (relative biological effectiveness, RBE^3^) was prescribed on the clinical target volume (CTV) median dose, and delivered to the planning target volume (PTV) in fractions of 1.8 Gy (RBE), with an RBE of 1.1 for PRT. The study complied with the Declaration of Helsinki, and was approved by the Ethics Review Board of Heidelberg University (protocol code: S-293/2022, Date of Approval: 25.04.2022).

### 2.2 Photon and carbon ion treatment planning

To evaluate the potential benefits of PRT and CIRT compared to VMAT, we retrospectively generated CIRT and VMAT plans in RayStation treatment planning system (TPS) (Version 2024B, RaySearch) using the original patient datasets and target volumes. Clinical proton planning aimed to ensure a CTV coverage of ≥95% of the prescribed dose, while respecting clinical dose goals, such as a D_max_ for the optic system of <54 Gy (RBE) or <52.2 Gy (RBE) in case of a higher individual risk. PRT plans were generated either in Siemens Syngo PT-Planning Software (Siemens Healthineers, Erlangen, Germany) or RayStation TPS (RaySearch Laboratories AB, Version 8 A-11B, Stockholm, Sweden). The overarching objective of the retrospective VMAT and CIRT planning was to reproduce the clinical OOI goals and objectives of the original PRT plan. Additional OOIs particularly relevant to our analysis were added according to the European Particle Therapy Network (EPTN) guidelines, but used only for reporting [21]. Beam arrangements followed institutional standards and were individualized based on target localization and anatomical considerations. Further details are provided in Supplementary Material B. All plans were fully reviewed and approved by radiation oncologists prior to analysis.

For CIRT, RBE-weighted dose calculations were performed using the local effect model I (LEM I) with an α/β of 2 Gy [22], [23]. All RBE calculations followed the clinical standards of our department. Consistent with routine clinical practice, PRT plans were initially planned with a scheme of 1.8 Gy (RBE) in 30 fractions. VMAT plans were generated using the same fractionation scheme. These smaller fraction doses, as used for PRT and VMAT, are not clinically validated for CIRT, as they are associated with higher RBE values and increased RBE modeling susceptibility [24]. Based on the established clinical experience at GSI, Darmstadt, CIRT was therefore planned using a scheme of 42 Gy (RBE) in 3 Gy (RBE) per fraction, corresponding to an equivalent dose in 2 Gy fractions (EQD2) of 52.5 Gy. To achieve the same EQD2 of 52.5 Gy for VMAT and PRT, these plans were normalized to a total dose of 54.9 Gy. For all modalities, the EQD2 values for OOIs met the tolerance dose goals set by the PRT plans.

### 2.3 Comparative evaluation of treatment plans

For comparison of dose exposure, the dose-volume histograms (DVHs) of target volumes (gross tumor volume (GTV), CTV and PTV) and selected OOI volumes were extracted from the TPS. For all comparisons, converted EQD2 was used.

Target coverage was analyzed using the following parameters: D_95%_ (minimum dose in 95% of the target volume), D_0.03cm3_ (minimum dose in 0.03 cm^3^ of the target volume), D_mean_ (mean dose), V_95%_ (volume receiving ≥95% of the planned dose), Homogeneity Index (HI), Conformity Index (CI). For the HI, D_5%_ and D_95%_ represent the minimum doses in 5% and 95% of the CTV, respectively. The optimal HI value is zero, with higher values indicating a less homogeneous plan [25].

(1) $$\mathrm{HI}=100\;x\;\frac{D5\%-D95\%}{\mathrm{DP}}$$

For CI, V_95%_ (CTV) and V_95%_ (Body) are the volumes of the CTV and body receiving ≥95% of the dose [26]. An optimal CI is close to 1, indicating a more conformal treatment with respect to normal tissue.

(2) $$\mathrm{CI}=100\;x\;\frac{V95\%\left(\mathrm{CTV}\right)2}{\mathrm{CTV}\cdot V95\%\left(\mathrm{Body}\right)}$$

A number of parameters derived from the DVHs were extracted for the specific OOI, including D_x%_ (minimum dose in x% of the OOI), D_0.03cm3_ (minimum dose in 0.03 cm^3^ of the OOI), D_mean_ (mean dose in the OOI), and V_xGy_ (volume of the OOI receiving ≥ x Gy). In paired organs, laterality was determined based on the primary tumor location, regardless of any subsequent bilateral tumor growth.

For all quantitative analyses, absolute and relative reductions between the Investigated modality (Inv. Mod.: protons (P) or carbon ions (C)) and the Reference modality (Ref. Mod.: photons (X)) were compiled.

The following equation was utilized for absolute reductions:

(3) $$\Delta_{\mathrm{abs}}=\mathrm{Inv}.\mathrm{Mod}.–\mathrm{Ref}.\mathrm{Mod}$$

The following equation was utilized for relative reductions:

(4) $$\Delta_{\mathrm{rel}}\left(P-X\right)=\left(\mathrm{Inv}.\mathrm{Mod}.–\mathrm{Ref}.\mathrm{Mod}.\right)/\left(\mathrm{Ref}.\mathrm{Mod}.\right)$$

When calculated dose-volume parameters in all three modalities were close to zero, relative reduction analysis was not applicable, and “-“ was used.

### 2.4 Model-based calculations of NTCPs and secondary neoplasms

To estimate potential adverse events of irradiation with these modalities, such as brain necrosis, hearing impairment and xerostomia, NTCPs were computed using multiple OOI-specific NTCP models. Details on these NTCP models are provided in Supplementary Table S1. For NTCP, the dose metrics were converted to EQD2.

Potentially elevated risks for radiation-induced CNS malignancies following VMAT, compared to PRT and CIRT, respectively, were estimated using risk ratios (RR). The RR is defined as the ratio of the excess absolute risk caused by photons, based on a carcinoma model [27], [28]. It can be approximated as the ratio of the organ equivalent dose (OED) for radiation-induced malignancies between two modalities [27], [28]:

(5) $$\mathrm{RR}=\mathrm{OED}\left(\mathrm{photon}\right)/\mathrm{OED}\left(\mathrm{proton}\right)\;$$

(6) $$\mathrm{RR}=\mathrm{OED}\left(\mathrm{photon}\right)/\mathrm{OED}\left(\mathrm{carbon}\;\mathrm{ion}\right)\;$$

The mathematical model of the OED is provided in Supplementary Material C.

### 2.5 Statistical analysis

Statistical analyses were conducted using MatLab (The MathWorks Inc., Version R2009b, Natick, MA, USA). Paired differences were assessed using the two-sided Wilcoxon signed-rank test. A *p*-value <0.05 was considered statistically significant.

## 3 Results

Median age at diagnosis was 52 years (range: 41–79). The median PTV and CTV were 119.0 cm^3^ (range: 62.1–464.0 cm^3^) and 67.1 cm^3^ (range: 35.3–336.7 cm^3^), respectively. No patient had prior radiotherapy, while five patients (56%) had undergone surgical resection before PRT. With a median follow-up of 44.0 months (range: 2–106 months), the estimated 3-year local PFS was 87.5%, and overall survival (OS) was 100.0%.

**Fig. 1:**
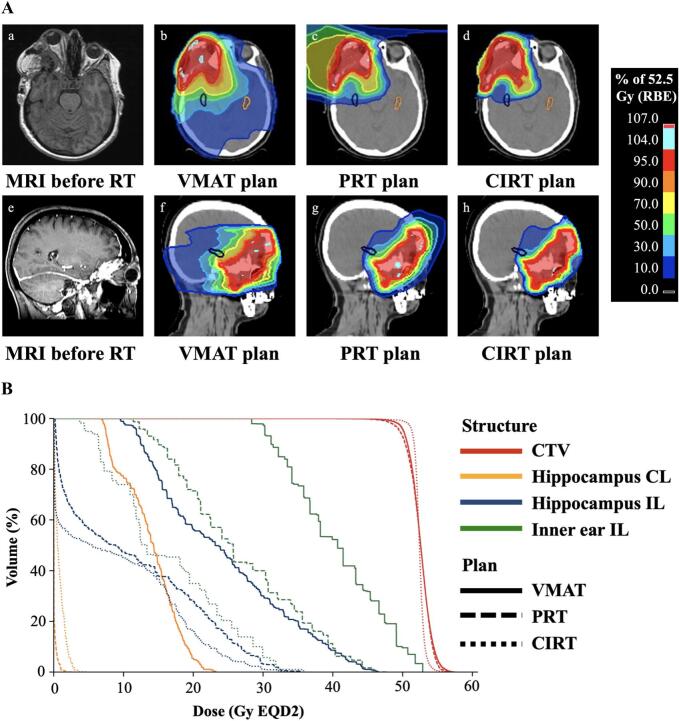
(A) Axial and sagittal treatment plans of a representative patient with SWM. (a) Axial T1-weighted MRI before the start of PRT. Axial (b) VMAT, (c) PRT, and (d) CIRT treatment plan. (e) Sagittal T1-weighted MRI before the start of PRT. Sagittal (f) VMAT, (g) PRT, and (h) CIRT treatment plan. Displayed are colored isodose lines as a percentage of the planned dose (52.5 Gy (EQD2)). Dose reductions were seen in PRT and CIRT plans compared to VMAT plans. (B) Comparison of exemplary DVH for selected OOIs, as well as for the CTV. The same exemplary patient as in Fig. 1 (A) is presented. Displayed are the CTV, as well as the ipsilateral and contralateral hippocampus, and the ipsilateral inner ear. DVH from VMAT, PRT, and CIRT plans are represented with solid, large-dashed, and small-dashed lines, respectively. All treatment modalities were planned to an EQD2 total dose of 52.5 Gy. The CTV D_50%_ corresponds to 52.57 Gy (EQD2) for the photon and proton plans and to 52.39 Gy (EQD2) for the carbon ion plan.

Comparison of target volume coverage revealed a lower HI with CIRT, indicating a more homogeneous dose distribution and a reduced maximum dose (D_0.03cm3_) within the target, compared to VMAT. PRT and CIRT plans revealed a CI value closer to 1, indicating improved conformity with respect to normal tissue, compared to VMAT. CTV dose-volume metrics are presented in Table 1, and detailed in Supplementary Table S2. Representative axial and sagittal MRI sequences, and corresponding treatment plans for all three treatment modalities are presented in Fig. 1 (A).

**Table 1:** Dose-volume parameters regarding the CTV between proton (P), photon (X), and carbon ion (C) plans.

CTV	Metric	Inv. Mod.	Mean	±	SD	Ref. Mod.	Mean	±	SD	∆abs Mean	±	∆abs SD	p-Value
CTV	V_95%_	P	90.9	±	6.6	X	92.1	±	6.6	−1.2	±	2.3	0.16
CTV	V_95%_	C	92.7	±	8.7	X	92.1	±	6.6	0.6	±	5.9	0.20
CTV	D_0.03cm3_	P	109.3	±	3.7	X	108.0	±	1.3	1.3	±	3.9	0.43
CTV	D_0.03cm3_	C	106.1	±	0.9	X	108.0	±	1.3	−1.9	±	1.3	0.00*
CTV	HI	P	10.2	±	3.1	X	10.3	±	1.8	−0.1	±	2.4	0.82
CTV	HI	C	7.0	±	3.3	X	10.3	±	1.8	−3.3	±	2.0	0.01*
CTV	CI	P	0.6	±	0.1	X	0.5	±	0.1	0.1	±	0.1	0.01*
CTV	CI	C	0.6	±	0.1	X	0.5	±	0.1	0.1	±	0.1	0.02*

Doses are expressed as a percentage of the planned dose; Volumes are expressed as a percentage of the CTV volume; V_x%_: volume receiving a minimum dose of x% of the planned dose; D_0.03cm_^3^: minimum dose received in 0.03 cm^3^ of the CTV; D_x%_: minimum dose received by x% of the volume; CTV: clinical target volume; HI: homogeneity index; CI: conformity index; SD: standard deviation; ∆abs: difference in absolute values between the Investigated modality (Inv. Mod.: Protons (P) or Carbon ions (C)) and the Reference modality (Ref. Mod.: Photons (X)); *: *p*-value <0.05; Reported values are rounded to one decimal place. The most pronounced dose reductions are indicated in bold font type.

Selected dose-volume parameters of OOIs with notable differences are presented in Table 2, and the complete overview of all OOIs is provided in Supplementary Table S3. Representative DVHs for selected OOIs and the CTV are presented in Fig. 1 (B). The most pronounced dose reductions were observed in cerebral structures, including brain, temporal lobes, and hippocampi, in ocular structures, and the inner ear.

**Table 2:** Dose-volume parameters for selected OOIs between proton (P), photon (X), and carbon ion (C) plans.

OOI	Metric	Inv. Mod.	Mean	±	SD	Ref. Mod.	Mean	±	SD	∆abs Mean	±	∆abs SD	p-Value
Brain	D_mean_	P	4.6	±	1.2	X	8.3	±	2.5	−3.7	±	2.4	0.00*
Brain	D_mean_	C	2.8	±	0.7	X	8.3	±	2.5	−5.5	±	2.3	0.00*
Eye CL	D_0.03cm_^3^	P	4.3	±	4.6	X	10.4	±	9.4	−6.1	±	7.1	0.02*
Eye CL	D_0.03cm_^3^	C	1.9	±	2.4	X	10.4	±	9.4	−8.6	±	7.4	0.01*
Eye IL	D_0.03cm_^3^	P	25.2	±	20.3	X	27.7	±	16.5	−2.5	±	6.5	0.36
Eye IL	D_0.03cm_^3^	C	20.7	±	22.4	X	27.7	±	16.5	−7.0	±	7.0	0.03*
Hippocampus bilateral	D_40%_	P	23.8	±	10.4	X	31.6	±	8.5	−7.8	±	6.8	0.02*
Hippocampus bilateral	D_40%_	C	16.9	±	8.3	X	31.6	±	8.5	−14.7	±	4.9	0.01*
Inner ear CL	D_mean_	P	14.4	±	9.9	X	19.2	±	13.4	−4.8	±	7.1	0.38
Inner ear CL	D_mean_	C	11.0	±	12.4	X	19.2	±	13.4	−8.2	±	2.8	0.01*
Inner ear IL	D_mean_	P	32.6	±	11.8	X	40.5	±	7.6	−7.9	±	7.0	0.02*
Inner ear IL	D_mean_	C	30.7	±	13.1	X	40.5	±	7.6	−9.8	±	8.0	0.01*
Lacrimal gland CL	D_mean_	P	1.8	±	2.2	X	6.4	±	6.8	−4.6	±	6.0	0.04*
Lacrimal gland CL	D_mean_	C	0.6	±	0.6	X	6.4	±	6.8	−5.8	±	6.5	0.00*
Lacrimal gland IL	D_mean_	P	13.3	±	15.6	X	19.0	±	14.6	−5.7	±	3.8	0.00*
Lacrimal gland IL	D_mean_	C	11.1	±	15.8	X	19.0	±	14.6	−7.8	±	4.7	0.00*
Lens CL	D_0.03cm_^3^	P	0.5	±	0.6	X	2.6	±	0.9	−2.0	±	1.2	0.00*
Lens CL	D_0.03cm_^3^	C	0.4	±	0.7	X	2.6	±	0.9	−2.1	±	0.9	0.00*
Lens IL	D_0.03cm_^3^	P	4.8	±	8.8	X	8.5	±	11.8	−3.7	±	4.4	0.01*
Lens IL	D_0.03cm_^3^	C	2.2	±	6.0	X	8.5	±	11.8	−6.4	±	6.8	0.00*
Temporal lobe CL	D_mean_	P	9.1	±	5.8	X	12.3	±	8.2	−3.3	±	5.4	0.25
Temporal lobe CL	D_mean_	C	5.2	±	4.6	X	12.3	±	8.2	−7.2	±	4.2	0.01*
Temporal lobe IL	D_mean_	P	15.3	±	6.0	X	24.5	±	3.8	−9.2	±	4.3	0.01*
Temporal lobe IL	D_mean_	C	13.4	±	4.7	X	24.5	±	3.8	−11.1	±	4.2	0.01*

Absolute values are expressed in Gy (EQD2); D_x%_: minimum dose in x% of the OOI; D_0.03cm_^3^: minimum dose in 0.03 cm^3^ of the OOI; D_mean_: mean dose in the OOI; CL: contralateral; IL: ipsilateral; SD: standard deviation; ∆abs: difference in absolute values between the Investigated modality (Inv. Mod.: Protons (P) or Carbon ions (C)) and the Reference modality (Ref. Mod.: Photons (X)); *: *p*-value 0.05; Reported values are rounded to one decimal place. The most pronounced dose reductions are indicated in bold font type.

For the brain, the mean dose was reduced by −3.7 Gy (EQD2) in PRT, and by −5.5 Gy (EQD2) in CIRT. Volume metrics (V_10Gy_ – V_50Gy_) showed relative reductions ranging from -24.4% (V_35Gy_) to −40.4% (V_10Gy_) in PRT, and from −37.4% (V_40Gy_) to −57.7% (V_10Gy_) in CIRT, compared with VMAT. Substantial sparing was observed for bilateral hippocampi. Within D_40%,_ absolute doses were reduced by −7.8 Gy (EQD2) for PRT, and by −14.7 Gy (EQD2) for CIRT. When analyzed by laterality, ipsilateral hippocampal sparing was significant in PRT, while both ipsilateral and contralateral hippocampal sparing was significant in CIRT. Within the ipsilateral temporal lobe, mean dose was reduced with PRT by −9.2 Gy (EQD2), and with CIRT by −11.1 Gy (EQD2), and also within the contralateral temporal lobe with CIRT by −7.2 Gy (EQD2).

For paired ocular structures (eye, lacrimal gland, and lens), significant dose reductions were achieved bilaterally with both PRT and CIRT. PRT reduced the absolute mean dose by −5.7 Gy (EQD2) in the ipsilateral lacrimal gland (relative: −48.7%), and by −4.6 Gy (EQD2) in the contralateral lacrimal gland (relative: 65.8%). With CIRT, the most pronounced reductions were observed in the eyes, with a mean maximum dose reduction of −7.0 Gy (EQD2) on the ipsilateral side and − 8.6 Gy (EQD2) on the contralateral side, and in the lacrimal gland, with a mean dose decrease of −7.8 Gy (EQD2) ipsilateral and − 5.8 Gy (EQD2) contralateral. Furthermore, dose exposure to the inner ear was reduced by particle therapy. For the ipsilateral inner ear, mean dose was reduced by −7.9 Gy (EQD2) in PRT, and by −9.8 Gy (EQD2) in CIRT. In the contralateral inner ear, CIRT reduced the mean dose by −8.2 Gy (EQD2).

**Fig. 2:**
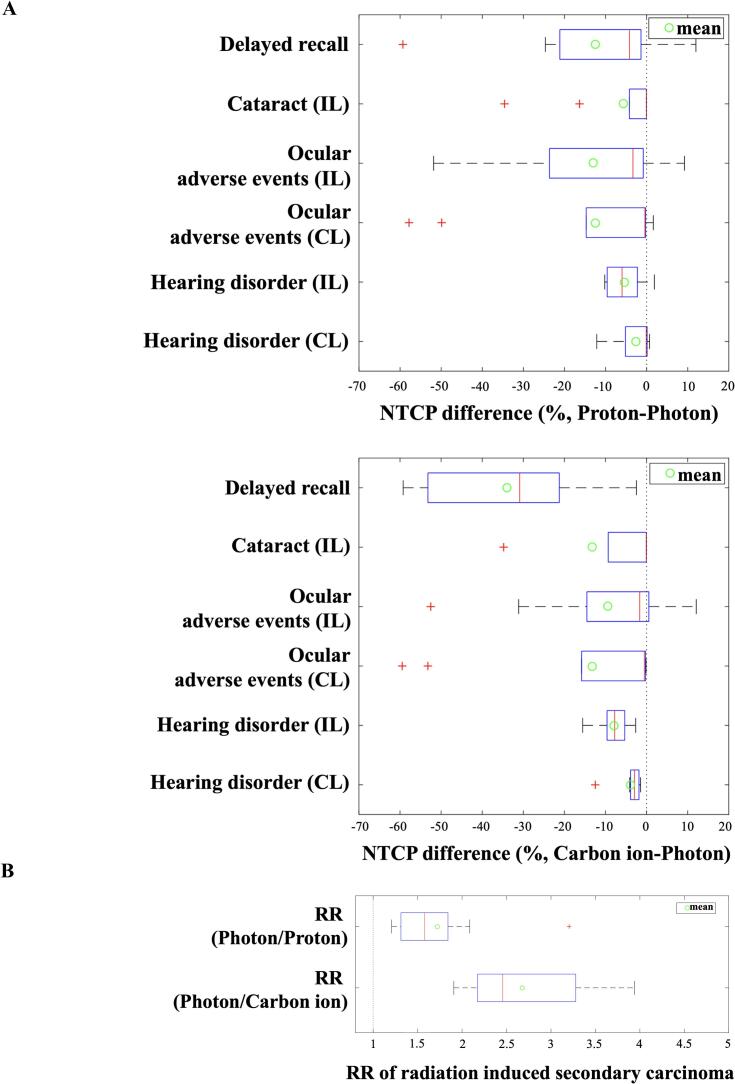
(A) Differences in NTCPs between PRT and VMAT, as well as CIRT and VMAT; (B) Differences in RRs for secondary CNS malignancies between PRT and VMAT, as well as CIRT and VMAT. ∆NTCP values are expressed as %.

Several NTCP models demonstrated significant differences in favor of particle therapy, with key findings displayed in Table 3 and Fig. 2 (A), and detailed in Supplementary Table S4. The most pronounced differences were observed for delayed recall, ipsilateral hearing loss and tinnitus, ipsilateral cataract, and contralateral ocular side effects. Notable differences were also observed for the risk of radiation-induced secondary malignancies.

**Table 3:** NTCPs for selected OOIs between proton (P), photon (X), and carbon ion (C) plans.

OOI/NTCP	Inv. Mod.	Mean	±	SD	Ref. Mod.	Mean	±	SD	∆abs Mean	±	∆abs SD	p-Value
Hippocampus bilateral: Delayed recall	P	79.6	±	32.0	X	92.2	±	13.5	−12.6	±	21.9	0.08
Hippocampus bilateral: Delayed recall	C	58.1	±	28.0	X	92.2	±	13.5	−34.0	±	20.0	0.01*
Inner Ear CL: Hearing loss	P	0.0	±	0.1	X	1.7	±	4.7	−1.6	±	4.6	0.50
Inner Ear CL: Hearing loss	C	0.1	±	0.2	X	1.7	±	4.7	−1.6	±	4.5	0.50
Inner Ear CL: side effect	P	8.1	±	4.4	X	10.8	±	8.3	−2.7	±	4.6	0.25
Inner Ear CL: side effect	C	6.9	±	4.9	X	10.8	±	8.3	−3.9	±	3.6	0.01*
Inner Ear IL: Hearing loss	P	5.2	±	10.6	X	8.5	±	13.8	−3.2	±	4.9	0.02*
Inner Ear IL: Hearing loss	C	5.1	±	10.3	X	8.5	±	13.8	−3.3	±	5.0	0.01*
Inner Ear IL: side effect	P	18.2	±	9.2	X	23.7	±	7.0	−5.5	±	4.5	0.02*
Inner Ear IL: side effect	C	15.7	±	8.6	X	23.7	±	7.0	−8.0	±	3.9	0.01*
Lacrimal gland CL: Ocular side effects	P	1.5	±	1.4	X	13.9	±	24.7	−12.4	±	23.6	0.05
Lacrimal gland CL: Ocular side effects	C	0.7	±	0.1	X	13.9	±	24.7	−13.2	±	24.5	0.00*
Lens IL: Cataract	P	9.4	±	27.8	X	15.1	±	33.8	−5.7	±	12.1	0.00*
Lens IL: Cataract	C	1.9	±	5.7	X	15.1	±	33.8	−13.2	±	28.5	0.00*

NTCPs are expressed in %; CL: contralateral; IL: ipsilateral; SD: standard deviation; ∆abs: difference in absolute values regarding a specific NTCP between the Investigated modality (Inv. Mod.: Protons (P) or Carbon ions (C)) and the Reference modality (Ref. Mod.: Photons (X)); *: *p*-value <0.05; Reported values are rounded to one decimal place. The most pronounced dose reductions are indicated in bold font type.

The estimated absolute probability of delayed recall was reduced by −34.0% with CIRT, compared to VMAT, with individual reductions of up to −59.2%. For the ipsilateral inner ear, PRT decreased the absolute risk of hearing loss and other side effects, such as tinnitus, by −3.2% and −5.5%, respectively. CIRT achieved absolute reductions in ipsilateral hearing loss of −3.3%, and other side effects of −8.0%, compared to VMAT. In the contralateral inner ear, CIRT decreased the absolute risk of other side effects, such as tinnitus, by −3.9% (relative: -34.9%). CIRT also reduced the absolute risk of side effects within the contralateral lacrimal gland by −13.2%, compared to VMAT. The absolute probability of cataract formation within the ipsilateral lens was reduced by −5.7% (relative: −64.0%) with PRT, and − 13.2% (relative: −86.2%) with CIRT. Other NTCP models demonstrated significant, though slight reductions, e.g., in brain and brainstem necrosis.

The mean estimated risk of radiation-induced secondary CNS malignancies of photons over protons was 1.7, with individual risks ranging from 1.2 to 3.2. Similarly, the mean estimated risk for photons over carbon ions was 2.7, with individual risks ranging from 1.9 to 3.9. Notable findings are displayed in Fig. 2 (B) and Supplementary Table S5.

## 4 Discussion

This comparative treatment planning study evaluated the dose distributions of VMAT, PRT and CIRT in nine patients with extensive SWMs treated at our institution. Particle therapy substantially reduced the mean dose within the brain, as well as in bilateral hippocampi and lenses, and the ipsilateral inner ear. NTCP-based estimations indicated lower risks of ipsilateral hearing impairment, cataract, and radiation-induced secondary CNS malignancies with particle therapy. These findings indicated that both PRT and CIRT may reduce radiation-induced side effects in extensive SWMs, compared to VMAT.

Gondi et al. identified 7.3 Gy (EQD2) to 40% of the bilateral hippocampi as a threshold associated with long-term impairment in delayed recall [16]. The ASCO-SNO-ASTRO guidelines recommend hippocampal sparing in whole-brain radiotherapy to preserve memory function and quality of life [29], [30]. This principle remains applicable in focal radiotherapy, particularly in patients with favorable prognosis. We observed an estimated absolute reduction of −34.0% in delayed recall with CIRT, compared to VMAT. The multicenter CANCER COG cohort study currently investigates the neurocognitive function in patients with WHO grade 1/2 SBMs, treated with either XRT or PRT [31].

Severe late effects of cranial irradiation include secondary malignancies. In a retrospective cohort of 426 patients (median age of 50 years) with pituitary adenoma, Minniti et al. reported a 20-year cumulative risk of secondary brain tumors of 2.4% [32]. Treatment was delivered with conventional external beam radiotherapy (76% receiving 40–50 Gy) between 1962 and 1994, using a technique far less precise than modern stereotactic methods [32]. In our assessment, the mean relative risk of secondary CNS malignancies was 1.7 for VMAT over PRT, and 2.7 for VMAT over CIRT. The applied models did not account for the generation of secondary neutrons. However, with active raster scanning used for this treatment, the additional integral dose from secondary neutrons was minimal compared to the dose sparing achieved with PRT, and was considered negligible [33], [34].

Given the limited availability of particle centers, appropriate resource allocation is critical [20]. Applying the treatment selection strategy proposed by Dutz et al., using a threshold of ∆NTCP >10% for at least one model-based adverse event, when compared to VMAT, all but one patient qualified for PRT and CIRT [35].

Heavy ions exhibit a higher linear energy transfer (LET) than protons and photons, potentially increasing radiobiological effectiveness [18]. Therefore, CIRT is primarily used for high-risk and re-irradiation cases. Ongoing initiatives, including the ReCare and ReCOG studies, aim to establish evidence-based dose guidance and clinical decision-making in re-irradiation scenarios [36], [37]. Emerging approaches, such as helium ions, may further reduce dose to OOIs in selected patients [38], [39]. Economically, particle therapy entails higher treatment costs than XRT [40], [41]. However, reduced long-term side effects may render particle therapy cost-effective in specific tumor entities and for selected patients with favorable prognoses and anatomical complexities [40], [41].

Several limitations must be acknowledged when interpreting our results. A constant RBE of 1.1 was applied for PRT; however, previous studies indicated that the RBE increases at the distal end of the beam, potentially raising the biological dose in adjacent tissues, which may introduce uncertainties in NTCP modeling [42]. For CIRT, RBE was calculated using LEM I with an α/β ratio of 2 Gy for all OOIs. A recent study on prostate cancer indicated that the high LET of carbon ions may lead to an RBE overestimation, underscoring the need for ongoing research to optimize RBE modeling [43].

The applied NTCP and secondary malignancy risk models merely provided theoretical estimations, but offered valuable insights into comparative profiles of side effects. Radiation-induced adverse events in SBMs are well-documented; selected publications are summarized in Supplementary Table S6. Previous studies indicated that radiation-induced cerebral contrast enhancement may be associated with increased average LET, particularly in regions distal to the Bragg Peak, as well as with patient comorbidities, rather than physical dose alone [44], [45]. Importantly, the applied brain necrosis model assumed an RBE of 1.1 for PRT and did not account for LET-dependent biological effects or heterogeneous dose distributions. Prospective trials are required to validate the clinical relevance, however, randomization between photons and protons poses ethical challenges.

All treatment plans were clinically reviewed and approved by radiation oncologists. However, beam arrangements and trade-offs between target coverage and OOI sparing were individualized for each patient and modality, introducing a potential interobserver bias and interpatient variability in OOI doses. A further methodological aspect concerns the evaluation of the PTV, particularly when comparing photon and particle treatment plans. A uniform, isotropic CTV-to-PTV margin across all modalities was used, whereas particle therapy is increasingly moving towards beam-specific PTVs or robust optimization on the CTV [46]. CIRT is primarily used for re-irradiation cases, where cumulative dose deposition is critical, and the steep dose gradient allows dose escalation while minimizing surrounding tissue exposure [47]. CIRT and PRT therefore represent different clinical settings, and direct comparison was not performed. Nevertheless, in selected cases, bimodal dose concepts might be a promising strategy, as demonstrated in the MARCIE trial [13].

Both PRT and CIRT exhibited superior dose distributions compared to VMAT, with significant sparing of brain tissue, bilateral hippocampi, lenses, and the ipsilateral inner ear. Consistently, NTCP modeling indicated a potential reduction of adverse events, such as hearing impairment and secondary CNS malignancies.

## CRediT authorship contribution statement

**Sophie Rauh:** Writing – original draft, Methodology, Investigation, Formal analysis, Conceptualization. **Maximilian Y. Deng:** Writing – original draft, Supervision, Resources, Project administration, Methodology, Investigation, Formal analysis, Conceptualization. **Inga Jessen:** Writing – review & editing, Resources, Methodology. **Lisa Seidel:** Writing – review & editing, Resources, Methodology. **Line Hoeltgen:** Writing – review & editing, Resources, Methodology. **Semi Harrabi:** Writing – review & editing, Resources, Methodology. **Filipa Baltazar:** Writing – review & editing, Resources, Methodology. **Thomas Haberer:** Writing – review & editing, Resources, Methodology. **Andrea Mairani:** Writing – review & editing, Resources, Methodology. **Klaus Herfarth:** Writing – review & editing, Resources, Methodology. **Jürgen Debus:** Writing – review & editing, Supervision, Resources, Methodology. **Thomas Tessonnier:** Writing – original draft, Validation, Supervision, Software, Resources, Project administration, Methodology, Investigation, Formal analysis, Conceptualization. **Laila König:** Writing – original draft, Supervision, Resources, Project administration, Methodology, Investigation, Formal analysis, Conceptualization.

## Ethics approval

The study was performed in accordance with the Declaration of Helsinki, and approval for the analysis was granted by the Ethics Review Board of Heidelberg University (protocol code: S-293/2022, Date of Approval: 25.04.2022). All procedures were performed in compliance with relevant laws and institutional guidelines. Patient confidentiality was maintained by anonymizing patient data to remove any identifying information.

## Funding sources

This research did not receive any specific grant from funding agencies in the public, commercial, or not-for-profit sectors.

## Declaration of competing interest

The authors declare the following financial interests/personal relationships which may be considered as potential competing interests: Maximilian Y. Deng reports a relationship with Else Kroner-Fresenius Foundation that includes: funding grants. Maximilian Y. Deng reports a relationship with German Consortium for Translational Cancer Research that includes: funding grants. Lisa Seidel reports a relationship with Mildred Scheel Doctoral Fellowship of the German Cancer Aid that includes: funding grants. Juergen Debus reports a relationship with RaySearch Laboratories AB that includes: consulting or advisory. Juergen Debus reports a relationship with Vision RT Ltd that includes: consulting or advisory. Juergen Debus reports a relationship with Merck Serono GmbH that includes: consulting or advisory. Juergen Debus reports a relationship with Siemens Healthcare GmbH that includes: consulting or advisory. Juergen Debus reports a relationship with PTW-Freiburg Dr. Pychlau GmbH that includes: consulting or advisory. Juergen Debus reports a relationship with Accuray Incorporated that includes: consulting or advisory. Juergen Debus reports a relationship with University Hospital Heidelberg Ion-Beam Therapy Center that includes: board membership and employment. Juergen Debus reports a relationship with University Hospital Heidelberg that includes: board membership. Juergen Debus reports a relationship with Intra-OP that includes: non-financial support. All other authors declare that they have no known competing financial interests or personal relationships that could have appeared to influence the work reported in this paper.

## Footnotes

3 The nomenclature Gy (RBE) refers to the definition provided in the International Commission on Radiation Units and Measurements (ICRU) Report 93.
