# Supplementary material for: Comparative treatment planning of photon, proton and carbon ion radiotherapy for sphenoid wing meningiomas
Source: Phys Imaging Radiat Oncol. 2026 Jun 13;39:101018. doi: 10.1016/j.phro.2026.101018 (PMC13316092; doi:10.1016/j.phro.2026.101018)
Supplement: Supplementary file 1 — Supplementary material [file mmc1.pdf]

Comparative treatment planning of photon, proton and carbon ion radiotherapy for sphenoid wing meningiomas

Supplementary Material A: Clinical Proton Treatment Planning and Delivery

For patient immobilization, a custom-manufactured, thermoplastic mask was employed. Treatment planning was based on neuroimaging, encompassing both CT and contrast-enhanced MRI. Gross tumor volume (GTV) was identified as the macroscopic tumor tissue visible on both radiological sequences. To include potential subclinical, microscopic tumor tissue, a margin of typically 1-5 mm was added to the GTV, defining the clinical target volume (CTV). A margin of 3 mm was added to the CTV, thus creating the planning target volume (PTV), compensating for physical inaccuracies and geometric uncertainties of the radiation beam. The organ of interest (OOIs), such as optic nerves and brain, were previously contoured. The principle of "as low as reasonably achievable" (ALARA) and normal tissue guidance according to QUANTEC and Emami et al. were applied [1, 2].

A total dose of 54 Gy (relative biological effectiveness, RBE [1]) was prescribed on the CTV median dose, and delivered to the PTV in fractions of 1.8 Gy (RBE) at Heidelberg Ion-Beam Therapy Center (HIT), which utilizes digital X-ray technology to provide two orthogonal images of the skull referenced to the treatment plan. Sub-millimeter accurate patient positioning was achieved using a robotic treatment table, capable of translations in six different directions. Treatment was delivered using active raster scanning technique in the horizontal treatment room under daily image-guidance.

Historically, Siemens Syngo PT-Planning Software (Siemens Healthineers, Erlangen, Germany) was used as the treatment planning system (TPS) at our institution, but was later replaced by RayStation TPS (RaySearch Laboratories AB, Version 2024B, Stockholm, Sweden). Proton radiotherapy (PRT) plans that had been planned with Siemens Syngo PT-Planning Software were then imported into RayStation TPS. For PRT, an RBE of 1.1 was utilized.

Supplementary Material B: Beam Arrangements in PRT and Carbon Ion Radiotherapy (CIRT)

In accordance with institutional practice, beam configurations were individualized based on target localization and anatomical considerations, including proximity to critical OOIs and RBE-related aspects in particle therapy. For PRT, target volumes were typically treated with opposed lateral beams. In selected cases, a third beam was incorporated to improve dose conformity and OOI sparing. Ipsilateral arrangements were generally avoided due to RBE-related considerations, particularly in distal, high linear energy transfer (LET) regions; however, in selected lateralized cases, where sufficient beam angulation was achievable,

[1] The nomenclature Gy (RBE) refers to the definition provided in the International Commission on Radiation Units and Measurements (ICRU) Report 93.

ipsilateral beams were applied. For CIRT, two beams were used, in accordance with clinical practice, employing opposed configurations for more centrally located targets and ipsilateral arrangements for more lateralized lesions.

Supplementary Material C: Calculation of the Organ Equivalent Dose (OED)

The OED for radiation-induced malignancies was calculated using the carcinoma model [3, 4]:

$$\mathrm{OED} = \frac{1}{V}\sum_{i} V_i \frac{\mathrm{e}^{-\alpha'_i D_i}}{\alpha'_i \, \mathrm{R}}\left(1 - 2\mathrm{R} + \mathrm{R}^2 \mathrm{e}^{\alpha'_i D_i} - (1-\mathrm{R})^2 \mathrm{e}^{-\frac{\alpha'_i \mathrm{R}}{1-\mathrm{R}} D_i}\right)$$

$$\text{with } \alpha'_i = \alpha + \beta \frac{D_i}{\mathrm{D_p}} \mathrm{d_T} \,.$$

α and β correspond to the parameters of the linear quadratic model, in this case $\frac{\alpha}{\beta} = 3$ Gy and $\alpha = 0.018$ Gy^{-1}. R represents the repair/repopulation parameter and R = 0.93. $\mathrm{D_p}$ is the planned dose to the CTV, and $\mathrm{d_T}$ is the planned dose per fraction. D_i and V_i are the respective dose and volume in each bin i of the dose-volume histogram (DVH) of the brain. V is the total volume of the brain. The total planned dose, number of fractions, and dose per fraction were used without the equivalent dose in 2 Gy fractions (EQD2) conversion to calculate the RR.

Supplementary Table S1. Models used for normal tissue complication probability (NTCP) estimations following radiotherapy.

TD_{50}: dose leading to a complication probability of 50%; gEUD: generalized equivalent uniform dose; IL: ipsilateral; CL: contralateral.

Organ of interest	Complication	Formula	Parameters	Publication
Brain (without CTV)	Necrosis Late/intermediate	$NTCP = (1 + (\frac{TD_{50}}{D})^{4\gamma})^{-1}$<br>$D = gEUD = [\sum_i (v_i D_i^{1/n})]^n$	*a = 9.0*<br>*n = 1/a*<br>*TD_{50} = 57.7 Gy*<br>*γ = 2.5*<br>*v_i, corresponding to a volume fraction of a structure receiving a dose D_i in 2 Gy equivalent dose using an α/β = 2.0 Gy*	Niyazi 2020 [5]
	Necrosis Late/severe	$NTCP = (1 + (\frac{TD_{50}}{Dmax})^{4\gamma})^{-1}$	*D_{max} = minimum dose received in 0.03 cm^3, in 2 Gy equivalent dose using an α/β = 0.96 Gy*<br>*TD_{50} = 109.0 Gy*<br>*γ = 2.8*	Bender 2012 [6]
Brainstem	Necrosis	$NTCP = (1 + (\frac{TD_{50}}{Dmax})^{4\gamma})^{-1}$	*D_{max} = minimum dose received in 0.03 cm^3, in 2 Gy equivalent dose using an α/β = 0.96 Gy*<br>*TD_{50} = 109.0 Gy*<br>*γ = 2.8*	Bender 2012 [6]
Chiasma<br>Optic nerve (IL/CL)	Blindness	$NTCP = \frac{1}{\sqrt{2\pi}} \int_{-\infty}^{t} e^{\frac{-x^2}{2}} dx$<br>$t = \frac{D - TD_{50}}{m \times TD_{50}}$<br>$D = gEUD = [\sum_i (v_i D_i^{1/n})]^n$	*a = 4.0*<br>*n = 1/a*<br>*TD_{50} = 65.0 Gy*<br>*m = 0.14*<br>*v_i, corresponding to a volume fraction of a structure receiving a dose D_i in 2 Gy equivalent dose using an α/β = 2.0 Gy*	Burman 1991 [7]
Inner ear (IL/CL)	Hearing disorder	$NTCP = (1 + e^{-\beta 0-\beta 1\times Dmean})^{-1}$	*D_{mean} = mean dose received in the structure in 2 Gy equivalent dose using an α/β = 2.0 Gy*<br>*β_0 = -3.3587*<br>*β_1 = 0.0517 Gy^{-1}*	Peuker 2022 [8]
	Hearing loss	$NTCP = (1 + (\frac{TD_{50}}{D})^{4\gamma})^{-1}$<br>$D = gEUD = [\sum_i (v_i D_i^{1/n})]^n$	*a = 6.4*<br>*n = 1/a*<br>*TD_{50} = 60.5 Gy*<br>*γ = 5.2*<br>*v_i, corresponding to a volume fraction of a structure receiving a dose D_i in 2 Gy equivalent dose using an α/β = 2 Gy*	De Marzi 2015 [9]
Hippocampus bilateral	Delayed recall	$NTCP = \frac{1}{\sqrt{2\pi}} \int_{-\infty}^{t} e^{\frac{-x^2}{2}} dx$<br>$t = \frac{D - TD_{50}}{m \times TD_{50}}$<br>$D = D40\%$	*$D_{40\%}$ = minimum dose received in 40% of the structure volume, in 2 Gy equivalent dose using an α/β = 2.0 Gy*<br>*TD_{50} = 14.88 Gy*<br>*m = 0.54*	Gondi 2013 [10]
Pituitary	Endocrine dysfunction	$NTCP = (1 + (\frac{TD_{50}}{D})^{4\gamma})^{-1}$<br>$D = gEUD = [\sum_i (v_i D_i^{1/n})]^n$	*n = 0.25*<br>*TD_{50} = 60.6 Gy*<br>*m = 0.15*<br>*α/β = 2.0 Gy*<br>*v_i, corresponding to a volume fraction of a structure receiving a dose D_i in 2 Gy equivalent dose using an α/β = 2.5 Gy*	De Marzi 2015 [9]
Lacrimal gland (IL/CL)	Ocular side effects	$NTCP = (1 + e^{-\beta 0-\beta 1\times Dmax})^{-1}$	*D_{max} = minimum dose received in 0.03 cm^3 of the structure in 2 Gy equivalent dose using an α/β = 2.0 Gy*<br>*β_0 = -5.174*<br>*β_1 = 0.205 Gy^{-1}*	Batth 2013 [11]
Lens (IL/CL)	Cataract	$NTCP = \frac{1}{\sqrt{2\pi}} \int_{-\infty}^{t} e^{\frac{-x^2}{2}} dx$<br>$t = \frac{D - TD_{50}}{m \times TD_{50}}$<br>$D = gEUD = [\sum_i (v_i D_i^{1/n})]^n$	*a = 3.33*<br>*n = 1/a*<br>*TD_{50} = 18.0 Gy*<br>*m = 0.27*<br>*v_i, corresponding to a volume fraction of a structure receiving a dose D_i in 2 Gy equivalent dose using an α/β = 2.0 Gy*	Burman 1991 [7]
Parotis (IL/CL)	Xerostomia	$NTCP = \frac{1}{\sqrt{2\pi}} \int_{-\infty}^{t} e^{\frac{-x^2}{2}} dx$<br>$t = \frac{D - TD_{50}}{m \times TD_{50}}$<br>$D = gEUD = [\sum_i (v_i D_i^{1/n})]^n$	*n = 1.0*<br>*TD_{50} = 39.9 Gy*<br>*m = 0.4*<br>*v_i, corresponding to a volume fraction of a structure receiving a dose D_i in 2 Gy equivalent dose using an α/β = 2.0 Gy*	Houweling 2010 [12]
Skin	Alopecia	$NTCP = (1 + e^{-\beta 0-\beta 1\times D5\%)})^{-1}$	*$D_{5\%}$ = minimum dose in 2 Gy equivalent dose using an α/β = 2.0 Gy received in 5% of the volume structure*<br>*β_0 = -1.33*<br>*β_1 = 0.08 Gy^{-1}*	Dutz 2019 [13]
	Erythema	$NTCP = (1 + e^{-\beta 0-\beta 1\times V35Gy)})^{-1}$	*V35Gy = volume of the structure in cm^3 receiving a minimum dose of 35 Gy in 2 Gy equivalent dose using an α/β = 2.0 Gy*	

			$\beta_0 = -1.54$<br>$\beta_1 = 0.06\ cm^{-3}$	

Supplementary Table S2. Dose-volume parameters regarding the GTV, CTV, and PTV between proton (P), photon (X), and carbon ion (C) plans.

Doses are expressed as a percentage of the planned dose; Volumes are expressed as a percentage of the target volume; Relative values are expressed in %; $V_{x\%}$: volume receiving a minimum dose of x% of the planned dose; $D_{0.03cm^3}$: minimum dose received in 0.03 cm^3 of the target volume; $D_{x\%}$: minimum dose received by x% of the volume; GTV: gross tumor volume; CTV: clinical target volume; PTV: planning target volume; HI: homogeneity index; CI: conformity index; SD: standard deviation; Δabs and Δrel: difference in absolute and relative values between the Investigated modality (Inv. Mod.: Protons (P) or Carbon ions (C)) and the Reference modality (Ref. Mod.: Photons (X)); *: p-value < 0.05; Reported values are rounded to two decimal places.

Volume	Metric	Com-parison	Inv. Mod.	Mean	±	SD	Ref. Mod.	Mean	±	SD	Δabs Mean	±	Δabs SD	Δrel Mean	±	Δrel SD	p-Value
GTV	$V_{95\%}$	P-X	P	92.59	±	7.27	X	92.99	±	7.12	-0.40	±	2.52	-0.40	±	2.78	1.00
GTV	$V_{95\%}$	C-X	C	93.65	±	8.32	X	92.99	±	7.12	0.65	±	5.69	0.81	±	6.50	0.20
GTV	$D_{95\%}$	P-X	P	94.51	±	2.66	X	94.64	±	1.65	-0.13	±	1.59	-0.14	±	1.69	0.91
GTV	$D_{95\%}$	C-X	C	95.53	±	2.97	X	94.64	±	1.65	0.89	±	1.74	0.92	±	1.83	0.25
CTV	$V_{95\%}$	P-X	P	90.94	±	6.63	X	92.14	±	6.55	-1.20	±	2.29	-1.28	±	2.49	0.16
CTV	$V_{95\%}$	C-X	C	92.73	±	8.73	X	92.14	±	6.55	0.59	±	5.94	0.67	±	6.70	0.20
CTV	D_{mean}	P-X	P	99.57	±	0.41	X	99.79	±	0.38	-0.22	±	0.29	-0.22	±	0.29	0.05
CTV	D_{mean}	C-X	C	99.35	±	0.60	X	99.79	±	0.38	-0.43	±	0.50	-0.43	±	0.50	0.02*
CTV	$D_{0.03cm^3}$	P-X	P	109.33	±	3.68	X	108.00	±	1.29	1.33	±	3.89	1.24	±	3.63	0.43
CTV	$D_{0.03cm^3}$	C-X	C	106.11	±	0.85	X	108.00	±	1.29	-1.90	±	1.25	-1.75	±	1.14	0.00*
CTV	$D_{95\%}$	P-X	P	93.68	±	2.30	X	94.29	±	1.58	-0.61	±	1.38	-0.66	±	1.48	0.25
CTV	$D_{95\%}$	C-X	C	95.09	±	2.80	X	94.29	±	1.58	0.80	±	1.74	0.84	±	1.84	0.36
CTV	HI	P-X	P	10.21	±	3.09	X	10.32	±	1.83	-0.11	±	2.39	-1.56	±	22.70	0.82
CTV	HI	C-X	C	7.03	±	3.31	X	10.32	±	1.83	-3.28	±	2.05	-34.22	±	24.47	0.01*
CTV	CI	P-X	P	0.61	±	0.10	X	0.53	±	0.12	0.07	±	0.07	16.39	±	20.73	0.01*
CTV	CI	C-X	C	0.58	±	0.09	X	0.53	±	0.12	0.05	±	0.06	11.63	±	18.46	0.02*
PTV	$V_{95\%}$	P-X	P	80.81	±	6.04	X	86.20	±	6.01	-5.38	±	5.23	-6.11	±	5.83	0.05
PTV	$V_{95\%}$	C-X	C	87.74	±	9.08	X	86.20	±	6.01	1.54	±	7.73	1.88	±	9.03	0.25
PTV	$D_{95\%}$	P-X	P	89.52	±	2.33	X	92.03	±	1.69	-2.52	±	2.69	-2.71	±	2.92	0.05
PTV	$D_{95\%}$	C-X	C	92.99	±	1.87	X	92.03	±	1.69	0.96	±	1.99	1.06	±	2.16	0.25
PTV	$D_{0.03cm^3}$	P-X	P	109.85	±	3.81	X	108.20	±	1.32	1.65	±	4.03	1.54	±	3.75	0.36
PTV	$D_{0.03cm^3}$	C-X	C	106.26	±	0.84	X	108.20	±	1.32	-1.94	±	1.32	-1.79	±	1.20	0.00*

Supplementary Table S3. Dose-volume parameters for all OOIs between proton (P), photon (X), and carbon ion (C) plans.

$D_{x\%}$: minimum dose in x% of the OOI; $D_{0.03cm^3}$: minimum dose in 0.03 cm³ of the OOI; D_{mean}: mean dose in the OOI; V_{xGy}: volume of the OOI receiving ≥ x Gy; Doses D_x are expressed in Gy (EQD2), and volumes in % of the structure volume, except for skin volumes (*), which are expressed in cm³; Relative values are expressed in %. CL: contralateral; IL: ipsilateral; SD: standard deviation; Δabs and Δrel: difference in absolute and relative values between the Investigated modality (Inv. Mod.: Protons (P) or Carbon ions (C)) and the Reference modality (Ref. Mod.: Photons (X)); *: p-value < 0.05; Reported values are rounded to two decimal places.

OOI	Metric	Com-parison	Inv. Mod.	Mean	±	SD	Ref. Mod.	Mean	±	SD	Δabs Mean	±	Δabs SD	Δrel Mean	±	Δrel SD	p-Value
Brain	$D_{0.03cm^3}$	P-X	P	55.08	±	0.88	X	55.20	±	1.22	-0.11	±	1.56	-0.16	±	2.89	0.82
Brain	$D_{0.03cm^3}$	C-X	C	54.95	±	0.53	X	55.20	±	1.22	-0.24	±	1.34	-0.40	±	2.51	0.25
Brain	D_{mean}	P-X	P	4.58	±	1.17	X	8.31	±	2.54	-3.74	±	2.37	-41.52	±	17.84	0.00*
Brain	D_{mean}	C-X	C	2.85	±	0.69	X	8.31	±	2.54	-5.46	±	2.31	-63.79	±	10.15	0.00*
Brain	V_{10Gy}	P-X	P	18.11	±	4.70	X	32.25	±	10.58	-14.15	±	9.16	-40.38	±	17.87	0.00*
Brain	V_{10Gy}	C-X	C	13.08	±	4.02	X	32.25	±	10.58	-19.17	±	9.00	-57.66	±	12.04	0.00*
Brain	V_{20Gy}	P-X	P	10.37	±	2.95	X	15.93	±	6.63	-5.55	±	4.23	-30.82	±	18.06	0.01*
Brain	V_{20Gy}	C-X	C	7.62	±	1.74	X	15.93	±	6.63	-8.31	±	5.16	-48.95	±	11.65	0.00*
Brain	V_{25Gy}	P-X	P	8.46	±	2.60	X	12.23	±	5.22	-3.78	±	3.03	-27.00	±	17.93	0.01*
Brain	V_{25Gy}	C-X	C	6.39	±	1.51	X	12.23	±	5.22	-5.84	±	3.88	-44.24	±	12.35	0.00*
Brain	V_{30Gy}	P-X	P	6.95	±	2.23	X	9.71	±	4.09	-2.75	±	2.20	-24.77	±	17.64	0.01*
Brain	V_{30Gy}	C-X	C	5.41	±	1.36	X	9.71	±	4.09	-4.30	±	2.85	-40.96	±	11.78	0.00*
Brain	V_{35Gy}	P-X	P	5.62	±	1.82	X	7.81	±	3.25	-2.19	±	1.77	-24.40	±	18.67	0.01*
Brain	V_{35Gy}	C-X	C	4.50	±	1.21	X	7.81	±	3.25	-3.31	±	2.13	-39.27	±	11.50	0.00*
Brain	V_{40Gy}	P-X	P	4.33	±	1.40	X	6.10	±	2.40	-1.77	±	1.36	-25.55	±	20.11	0.01*
Brain	V_{40Gy}	C-X	C	3.67	±	1.08	X	6.10	±	2.40	-2.43	±	1.41	-37.42	±	10.88	0.00*
Brain	V_{50Gy}	P-X	P	1.25	±	0.34	X	1.87	±	0.67	-0.62	±	0.60	-25.61	±	35.70	0.01*
Brain	V_{50Gy}	C-X	C	1.54	±	0.57	X	1.87	±	0.67	-0.32	±	0.56	-13.35	±	23.84	0.20
Brainstem	$D_{0.03cm^3}$	P-X	P	52.46	±	0.69	X	51.00	±	0.72	1.46	±	1.00	2.88	±	2.00	0.00*
Brainstem	$D_{0.03cm^3}$	C-X	C	50.88	±	0.97	X	51.00	±	0.72	-0.12	±	1.33	-0.21	±	2.63	0.57
Brainstem	$D_{2\%}$	P-X	P	50.34	±	1.93	X	49.94	±	1.06	0.41	±	1.43	0.81	±	2.91	0.36
Brainstem	$D_{2\%}$	C-X	C	49.39	±	1.64	X	49.94	±	1.06	-0.54	±	1.21	-1.09	±	2.46	0.36
Brainstem	D_{mean}	P-X	P	23.17	±	9.07	X	31.28	±	6.74	-8.10	±	4.65	-28.06	±	20.86	0.01*
Brainstem	D_{mean}	C-X	C	16.18	±	8.43	X	31.28	±	6.74	-15.10	±	4.48	-50.69	±	19.88	0.00*
Chiasma	$D_{0.03cm^3}$	P-X	P	49.06	±	1.39	X	48.82	±	0.93	0.24	±	1.22	0.50	±	2.56	0.57
Chiasma	$D_{0.03cm^3}$	C-X	C	48.11	±	1.34	X	48.82	±	0.93	-0.72	±	1.24	-1.46	±	2.59	0.10
Chiasma	D_{mean}	P-X	P	40.81	±	7.53	X	42.92	±	6.51	-2.10	±	3.75	-4.92	±	8.75	0.16
Chiasma	D_{mean}	C-X	C	37.79	±	10.17	X	42.92	±	6.51	-5.13	±	5.36	-13.32	±	14.59	0.01*
Eye CL	$D_{0.03cm^3}$	P-X	P	4.31	±	4.65	X	10.45	±	9.41	-6.14	±	7.08	-54.79	±	58.09	0.02*
Eye CL	$D_{0.03cm^3}$	C-X	C	1.87	±	2.36	X	10.45	±	9.41	-8.58	±	7.36	-84.54	±	13.53	0.01*
Eye CL	D_{mean}	P-X	P	1.09	±	1.50	X	3.85	±	3.68	-2.76	±	3.48	-58.72	±	59.50	0.02*
Eye CL	D_{mean}	C-X	C	0.56	±	0.76	X	3.85	±	3.68	-3.29	±	3.23	-76.31	±	33.30	0.01*
Eye IL	$D_{0.03cm^3}$	P-X	P	25.17	±	20.30	X	27.67	±	16.52	-2.51	±	6.54	-21.49	±	43.58	0.36
Eye IL	$D_{0.03cm^3}$	C-X	C	20.68	±	22.42	X	27.67	±	16.52	-7.00	±	6.97	-46.24	±	44.23	0.03*
Eye IL	D_{mean}	P-X	P	8.28	±	10.07	X	13.49	±	12.35	-5.21	±	3.47	-54.23	±	30.65	0.00*
Eye IL	D_{mean}	C-X	C	5.09	±	7.37	X	13.49	±	12.35	-8.40	±	5.34	-78.70	±	24.06	0.00*
Hippocampus bilateral	$D_{0.03cm^3}$	P-X	P	49.65	±	7.48	X	51.58	±	3.23	-1.93	±	4.48	-4.23	±	10.09	0.38

Hippocampus bilateral	$D_{0.03cm^3}$	C-X	C	50.19 ± 8.91	X	51.58 ± 3.23	-1.39 ± 5.95	-3.35 ± 13.38	0.55
Hippocampus bilateral	$D_{2\%}$	P-X	P	48.82 ± 7.96	X	50.97 ± 3.82	-2.15 ± 4.45	-4.83 ± 10.46	0.15
Hippocampus bilateral	$D_{2\%}$	C-X	C	49.30 ± 9.62	X	50.97 ± 3.82	-1.67 ± 6.18	-4.10 ± 14.50	1.00
Hippocampus bilateral	$D_{40\%}$	P-X	P	23.77 ± 10.41	X	31.59 ± 8.46	-7.83 ± 6.81	-27.16 ± 33.50	0.02*
Hippocampus bilateral	$D_{40\%}$	C-X	C	16.86 ± 8.33	X	31.59 ± 8.46	-14.73 ± 4.92	-48.97 ± 21.65	0.01*
Hippocampus bilateral	D_{mean}	P-X	P	19.32 ± 6.50	X	24.13 ± 5.16	-4.81 ± 6.71	-18.87 ± 31.17	0.15
Hippocampus bilateral	D_{mean}	C-X	C	13.13 ± 4.72	X	24.13 ± 5.16	-11.00 ± 5.62	-45.02 ± 20.37	0.01*
Hippocampus bilateral	V_{20Gy}	P-X	P	44.54 ± 14.35	X	56.47 ± 18.40	-11.94 ± 17.50	-18.44 ± 29.54	0.15
Hippocampus bilateral	V_{20Gy}	C-X	C	33.45 ± 13.34	X	56.47 ± 18.40	-23.03 ± 21.10	-38.05 ± 29.03	0.01*
Hippocampus CL	$D_{0.03cm^3}$	P-X	P	26.78 ± 18.06	X	28.45 ± 18.03	-1.68 ± 10.65	14.47 ± 79.59	1.00
Hippocampus CL	$D_{0.03cm^3}$	C-X	C	21.71 ± 21.84	X	28.45 ± 18.03	-6.74 ± 7.61	-36.86 ± 34.70	0.05
Hippocampus CL	$D_{2\%}$	P-X	P	26.67 ± 17.88	X	28.43 ± 18.03	-1.76 ± 10.65	14.78 ± 80.63	0.95
Hippocampus CL	$D_{2\%}$	C-X	C	21.71 ± 21.77	X	28.43 ± 18.03	-6.73 ± 7.59	-36.61 ± 34.56	0.05
Hippocampus CL	D_{mean}	P-X	P	13.76 ± 9.71	X	16.44 ± 12.49	-2.68 ± 6.84	7.76 ± 65.00	0.31
Hippocampus CL	D_{mean}	C-X	C	6.91 ± 7.22	X	16.44 ± 12.49	-9.53 ± 6.35	-63.79 ± 20.73	0.01*
Hippocampus CL	V_{20Gy}	P-X	P	22.83 ± 33.93	X	30.77 ± 38.86	-7.94 ± 20.99	-15.59 ± 69.11	0.44
Hippocampus CL	V_{20Gy}	C-X	C	12.67 ± 20.26	X	30.77 ± 38.86	-18.10 ± 24.17	-41.08 ± 40.72	0.06
Hippocampus IL	$D_{0.03cm^3}$	P-X	P	47.07 ± 8.40	X	50.33 ± 4.22	-3.25 ± 4.70	-7.08 ± 10.43	0.15
Hippocampus IL	$D_{0.03cm^3}$	C-X	C	46.61 ± 10.68	X	50.33 ± 4.22	-3.71 ± 6.60	-8.42 ± 15.00	0.38
Hippocampus IL	$D_{2\%}$	P-X	P	46.85 ± 8.57	X	50.34 ± 4.18	-3.48 ± 4.84	-7.57 ± 10.70	0.15
Hippocampus IL	$D_{2\%}$	C-X	C	46.44 ± 10.36	X	50.34 ± 4.18	-3.89 ± 6.27	-8.73 ± 14.19	0.31
Hippocampus IL	D_{mean}	P-X	P	26.38 ± 12.33	X	33.63 ± 8.28	-7.25 ± 6.39	-24.79 ± 25.28	0.04*
Hippocampus IL	D_{mean}	C-X	C	22.23 ± 12.59	X	33.63 ± 8.28	-11.40 ± 6.01	-38.13 ± 26.88	0.01*
Hippocampus IL	V_{20Gy}	P-X	P	64.91 ± 29.83	X	80.19 ± 15.62	-15.28 ± 20.34	-21.48 ± 29.51	0.11
Hippocampus IL	V_{20Gy}	C-X	C	52.66 ± 33.75	X	80.19 ± 15.62	-27.53 ± 23.34	-38.01 ± 34.98	0.01*
Inner ear CL	$D_{0.03cm^3}$	P-X	P	23.36 ± 17.78	X	25.77 ± 12.68	-2.42 ± 6.80	-18.71 ± 36.16	0.31
Inner ear CL	$D_{0.03cm^3}$	C-X	C	20.88 ± 18.08	X	25.77 ± 12.68	-4.89 ± 6.04	-31.77 ± 35.58	0.08
Inner ear CL	D_{mean}	P-X	P	14.44 ± 9.91	X	19.21 ± 13.42	-4.76 ± 7.12	-19.52 ± 36.62	0.38
Inner ear CL	D_{mean}	C-X	C	10.96 ± 12.43	X	19.21 ± 13.42	-8.25 ± 2.82	-54.60 ± 25.42	0.01*
Inner ear IL	$D_{0.03cm^3}$	P-X	P	48.50 ± 7.17	X	49.99 ± 5.93	-1.49 ± 4.61	-2.90 ± 9.44	0.55
Inner ear IL	$D_{0.03cm^3}$	C-X	C	45.85 ± 9.31	X	49.99 ± 5.93	-4.14 ± 7.94	-8.09 ± 16.30	0.15
Inner ear IL	D_{mean}	P-X	P	32.58 ± 11.80	X	40.52 ± 7.55	-7.94 ± 6.98	-20.76 ± 19.41	0.02*
Inner ear IL	D_{mean}	C-X	C	30.68 ± 13.09	X	40.52 ± 7.55	-9.84 ± 8.01	-26.02 ± 20.86	0.01*
Lacrimal gland CL	$D_{0.03cm^3}$	P-X	P	3.31 ± 4.06	X	9.06 ± 10.31	-5.75 ± 7.56	-62.82 ± 63.30	0.04*
Lacrimal gland CL	$D_{0.03cm^3}$	C-X	C	0.90 ± 0.99	X	9.06 ± 10.31	-8.16 ± 9.52	-85.57 ± 18.78	0.00*
Lacrimal gland CL	D_{mean}	P-X	P	1.77 ± 2.24	X	6.36 ± 6.75	-4.59 ± 6.02	-65.78 ± 69.02	0.04*
Lacrimal gland CL	D_{mean}	C-X	C	0.57 ± 0.60	X	6.36 ± 6.75	-5.79 ± 6.47	-86.61 ± 19.10	0.00*
Lacrimal gland CL	V_{30Gy}	P-X	P	0.01 ± 0.00	X	0.15 ± 0.42	-0.14 ± 0.42	-11.02 ± 33.07	1.00
Lacrimal gland CL	V_{30Gy}	C-X	C	0.01 ± 0.00	X	0.15 ± 0.42	-0.14 ± 0.42	-11.02 ± 33.07	1.00
Lacrimal gland IL	$D_{0.03cm^3}$	P-X	P	18.64 ± 18.28	X	26.13 ± 15.48	-7.49 ± 6.73	-44.88 ± 41.81	0.01*
Lacrimal gland IL	$D_{0.03cm^3}$	C-X	C	18.70 ± 20.41	X	26.13 ± 15.48	-7.44 ± 9.17	-48.34 ± 51.00	0.04*
Lacrimal gland IL	D_{mean}	P-X	P	13.29 ± 15.61	X	18.97 ± 14.59	-5.68 ± 3.83	-48.69 ± 40.00	0.00*
Lacrimal gland IL	D_{mean}	C-X	C	11.13 ± 15.76	X	18.97 ± 14.59	-7.83 ± 4.69	-61.64 ± 40.70	0.00*

Lacrimal gland IL	V_{30Gy}	P-X	P	17.80 ± 36.34	X	19.96 ± 37.48	-2.16 ± 4.53	10.39 ± 97.97	0.31
Lacrimal gland IL	V_{30Gy}	C-X	C	17.58 ± 33.40	X	19.96 ± 37.48	-2.38 ± 10.66	97.67 ± 308.97	1.00
Lens CL	$D_{0.03cm^3}$	P-X	P	0.53 ± 0.64	X	2.56 ± 0.94	-2.03 ± 1.16	-76.71 ± 32.98	0.00*
Lens CL	$D_{0.03cm^3}$	C-X	C	0.45 ± 0.71	X	2.56 ± 0.94	-2.11 ± 0.88	-83.92 ± 25.72	0.00*
Lens CL	D_{mean}	P-X	P	0.41 ± 0.50	X	2.30 ± 0.94	-1.89 ± 1.03	-81.26 ± 25.87	0.00*
Lens CL	D_{mean}	C-X	C	0.38 ± 0.62	X	2.30 ± 0.94	-1.91 ± 0.87	-84.90 ± 25.23	0.00*
Lens IL	$D_{0.03cm^3}$	P-X	P	4.80 ± 8.84	X	8.52 ± 11.83	-3.72 ± 4.42	-62.27 ± 34.78	0.01*
Lens IL	$D_{0.03cm^3}$	C-X	C	2.15 ± 5.97	X	8.52 ± 11.83	-6.37 ± 6.79	-92.34 ± 15.68	0.00*
Lens IL	D_{mean}	P-X	P	3.77 ± 6.92	X	7.58 ± 10.29	-3.80 ± 4.36	-66.72 ± 31.23	0.00*
Lens IL	D_{mean}	C-X	C	1.24 ± 3.42	X	7.58 ± 10.29	-6.33 ± 7.32	-94.74 ± 10.28	0.00*
Optic nerve CL	$D_{0.03cm^3}$	P-X	P	45.26 ± 10.05	X	44.57 ± 9.94	0.69 ± 1.68	1.58 ± 3.66	0.43
Optic nerve CL	$D_{0.03cm^3}$	C-X	C	43.54 ± 13.51	X	44.57 ± 9.94	-1.03 ± 4.01	-6.08 ± 20.11	0.57
Optic nerve CL	D_{mean}	P-X	P	22.22 ± 13.46	X	23.35 ± 13.41	-1.13 ± 5.89	-3.31 ± 27.28	1.00
Optic nerve CL	D_{mean}	C-X	C	17.60 ± 14.44	X	23.35 ± 13.41	-5.75 ± 3.67	-34.49 ± 25.10	0.01*
Optic nerve IL	$D_{0.03cm^3}$	P-X	P	49.97 ± 2.07	X	49.76 ± 2.19	0.22 ± 1.09	0.47 ± 2.25	0.43
Optic nerve IL	$D_{0.03cm^3}$	C-X	C	49.39 ± 1.91	X	49.76 ± 2.19	-0.36 ± 1.35	-0.68 ± 2.66	0.50
Optic nerve IL	D_{mean}	P-X	P	39.57 ± 8.97	X	41.33 ± 7.79	-1.76 ± 2.15	-4.81 ± 6.45	0.04*
Optic nerve IL	D_{mean}	C-X	C	38.02 ± 10.40	X	41.33 ± 7.79	-3.31 ± 2.87	-9.33 ± 8.88	0.00*
Parotis CL	$D_{0.03cm^3}$	P-X	P	12.95 ± 17.89	X	14.99 ± 15.68	-2.04 ± 4.75	-35.03 ± 43.33	0.13
Parotis CL	$D_{0.03cm^3}$	C-X	C	10.48 ± 17.95	X	14.99 ± 15.68	-4.51 ± 3.80	-57.67 ± 33.72	0.01*
Parotis CL	D_{mean}	P-X	P	2.60 ± 5.92	X	3.95 ± 4.31	-1.34 ± 1.83	-65.79 ± 41.46	0.10
Parotis CL	D_{mean}	C-X	C	1.94 ± 3.69	X	3.95 ± 4.31	-2.00 ± 0.93	-64.64 ± 20.16	0.00*
Parotis IL	$D_{0.03cm^3}$	P-X	P	22.58 ± 14.41	X	21.56 ± 15.09	1.01 ± 6.75	15.69 ± 40.15	0.57
Parotis IL	$D_{0.03cm^3}$	C-X	C	26.78 ± 12.59	X	21.56 ± 15.09	5.21 ± 11.41	70.78 ± 117.80	0.20
Parotis IL	D_{mean}	P-X	P	4.37 ± 6.63	X	4.30 ± 5.82	0.06 ± 2.91	20.13 ± 63.75	0.82
Parotis IL	D_{mean}	C-X	C	4.78 ± 7.43	X	4.30 ± 5.82	0.47 ± 2.89	21.50 ± 84.32	0.91
Pituitary	$D_{0.03cm^3}$	P-X	P	52.43 ± 1.45	X	53.66 ± 1.36	-1.23 ± 2.35	-2.21 ± 4.35	0.31
Pituitary	$D_{0.03cm^3}$	C-X	C	53.06 ± 1.12	X	53.66 ± 1.36	-0.60 ± 1.53	-1.07 ± 2.89	0.38
Pituitary	D_{mean}	P-X	P	50.37 ± 2.16	X	51.70 ± 1.41	-1.33 ± 2.18	-2.54 ± 4.25	0.15
Pituitary	D_{mean}	C-X	C	51.30 ± 1.44	X	51.70 ± 1.41	-0.41 ± 1.45	-0.76 ± 2.83	0.55
Skin	$D_{0.03cm^3}$	P-X	P	32.93 ± 18.40	X	36.09 ± 16.41	-3.16 ± 4.58	-12.03 ± 17.62	0.07
Skin	$D_{0.03cm^3}$	C-X	C	37.41 ± 13.37	X	36.09 ± 16.41	1.32 ± 7.72	12.68 ± 38.85	1.00
Skin	$D_{5\%}$	P-X	P	14.75 ± 14.03	X	15.64 ± 11.53	-0.89 ± 2.91	-13.61 ± 16.51	0.20
Skin	$D_{5\%}$	C-X	C	13.78 ± 14.90	X	15.64 ± 11.53	-1.85 ± 4.42	-25.07 ± 34.73	0.20
Skin	V_{10Gy*}	P-X	P	31.26 ± 32.31	X	54.84 ± 58.36	-23.59 ± 28.46	-45.57 ± 20.31	0.00*
Skin	V_{10Gy*}	C-X	C	33.44 ± 24.87	X	54.84 ± 58.36	-21.40 ± 35.26	-14.14 ± 39.18	0.07
Skin	V_{35Gy*}	P-X	P	7.26 ± 13.16	X	5.92 ± 10.86	1.34 ± 2.70	12.48 ± 36.13	0.25
Skin	V_{35Gy*}	C-X	C	7.25 ± 12.33	X	5.92 ± 10.86	1.33 ± 2.66	18.45 ± 60.46	0.25
Temporal lobe CL	$D_{0.03cm^3}$	P-X	P	50.93 ± 8.11	X	53.29 ± 3.03	-2.35 ± 6.21	-4.71 ± 12.33	0.46
Temporal lobe CL	$D_{0.03cm^3}$	C-X	C	52.37 ± 4.11	X	53.29 ± 3.03	-0.91 ± 2.91	-1.70 ± 5.78	0.46
Temporal lobe CL	D_{mean}	P-X	P	9.08 ± 5.76	X	12.35 ± 8.19	-3.26 ± 5.42	-11.49 ± 48.80	0.25
Temporal lobe CL	D_{mean}	C-X	C	5.16 ± 4.64	X	12.35 ± 8.19	-7.19 ± 4.23	-61.29 ± 16.28	0.01*
Temporal lobe CL	V_{10Gy}	P-X	P	31.97 ± 22.90	X	41.25 ± 29.43	-9.28 ± 23.84	9.11 ± 91.75	0.46
Temporal lobe CL	V_{10Gy}	C-X	C	20.96 ± 21.81	X	41.25 ± 29.43	-20.28 ± 16.70	-53.57 ± 24.59	0.01*

Temporal lobe CL	V_{20Gy}	P-X	P	14.00 ± 16.90	X	20.99 ± 20.65	-6.99 ± 6.36	-34.12 ± 27.48	0.02*
Temporal lobe CL	V_{20Gy}	C-X	C	10.88 ± 12.73	X	20.99 ± 20.65	-10.11 ± 8.72	-52.84 ± 17.14	0.01*
Temporal lobe CL	V_{25Gy}	P-X	P	11.49 ± 15.27	X	16.60 ± 18.49	-5.10 ± 4.76	-38.68 ± 29.04	0.01*
Temporal lobe CL	V_{25Gy}	C-X	C	8.95 ± 11.13	X	16.60 ± 18.49	-7.65 ± 7.58	-51.17 ± 15.46	0.01*
Temporal lobe IL	$D_{0.03cm^3}$	P-X	P	55.32 ± 0.94	X	55.45 ± 0.69	-0.13 ± 1.23	-0.23 ± 2.22	0.84
Temporal lobe IL	$D_{0.03cm^3}$	C-X	C	54.96 ± 0.39	X	55.45 ± 0.69	-0.49 ± 0.81	-0.88 ± 1.46	0.15
Temporal lobe IL	D_{mean}	P-X	P	15.34 ± 5.97	X	24.52 ± 3.83	-9.18 ± 4.29	-38.12 ± 19.15	0.01*
Temporal lobe IL	D_{mean}	C-X	C	13.37 ± 4.68	X	24.52 ± 3.83	-11.15 ± 4.23	-45.46 ± 16.41	0.01*
Temporal lobe IL	V_{10Gy}	P-X	P	55.64 ± 20.19	X	88.39 ± 7.78	-32.75 ± 18.30	-37.35 ± 20.77	0.01*
Temporal lobe IL	V_{10Gy}	C-X	C	53.00 ± 15.35	X	88.39 ± 7.78	-35.38 ± 13.60	-40.29 ± 15.32	0.01*
Temporal lobe IL	V_{20Gy}	P-X	P	34.11 ± 15.41	X	55.39 ± 13.50	-21.29 ± 11.44	-38.36 ± 25.61	0.02*
Temporal lobe IL	V_{20Gy}	C-X	C	32.12 ± 10.55	X	55.39 ± 13.50	-23.27 ± 12.16	-40.29 ± 21.30	0.02*
Temporal lobe IL	V_{25Gy}	P-X	P	27.18 ± 11.32	X	43.04 ± 11.97	-15.86 ± 8.14	-36.50 ± 23.27	0.02*
Temporal lobe IL	V_{25Gy}	C-X	C	26.74 ± 8.53	X	43.04 ± 11.97	-16.30 ± 10.13	-35.53 ± 22.70	0.02*

Supplementary Table S4. Normal tissue complication probabilities (NTCPs) for selected OOIs between proton (P), photon (X), and carbon ion (C) plans.

NTCPs are expressed in %. CTV: clinical target volume; CL: contralateral; IL: ipsilateral; SD: standard deviation; Δabs and Δrel: difference in absolute and relative values regarding a specific NTCP between the Investigated modality (Inv. Mod.: Protons (P) or Carbon ions (C)) and the Reference modality (Ref. Mod.: Photons (X)); *: p-value < 0.05; Reported values are rounded to two decimal places.

OOI/NTCP	Com- parison	Inv. Mod.	Mean	±	SD	Ref. Mod.	Mean	±	SD	Δabs Mean	±	Δabs SD	Δrel Mean	±	Δrel SD	p- Value
Brain-CTV: Necrosis-Intermediate/late	P-X	P	0.55	±	0.18	X	0.84	±	0.32	-0.29	±	0.23	-28.61	±	29.46	0.01*
Brain-CTV: Necrosis-Intermediate/late	C-X	C	0.56	±	0.20	X	0.84	±	0.32	-0.28	±	0.17	-31.09	±	13.27	0.00*
Brain-CTV: Necrosis-Severe/late	P-X	P	0.04	±	0.01	X	0.05	±	0.01	0.00	±	0.01	3.68	±	41.17	0.73
Brain-CTV: Necrosis-Severe/late	C-X	C	0.11	±	0.01	X	0.05	±	0.01	0.06	±	0.02	151.70	±	99.95	0.00*
Brainstem: Necrosis	P-X	P	0.02	±	0.00	X	0.02	±	0.00	0.01	±	0.01	46.46	±	37.86	0.00*
Brainstem: Necrosis	C-X	C	0.04	±	0.01	X	0.02	±	0.00	0.03	±	0.01	162.52	±	96.68	0.00*
Chiasma: Blindness	P-X	P	1.48	±	1.21	X	1.68	±	1.09	-0.20	±	0.87	-16.50	±	54.11	0.50
Chiasma: Blindness	C-X	C	1.26	±	1.03	X	1.68	±	1.09	-0.42	±	0.62	-35.66	±	40.33	0.07
Hippocampus bilateral: Delayed recall	P-X	P	79.60	±	31.99	X	92.19	±	13.49	-12.59	±	21.90	-16.68	±	33.46	0.08
Hippocampus bilateral: Delayed recall	C-X	C	58.14	±	28.04	X	92.19	±	13.49	-34.05	±	19.96	-39.34	±	27.69	0.01*
Inner Ear CL: Hearing loss	P-X	P	0.05	±	0.13	X	1.67	±	4.71	-1.63	±	4.57	-1.45	±	126.86	0.50
Inner Ear CL: Hearing loss	C-X	C	0.08	±	0.23	X	1.67	±	4.71	-1.59	±	4.48	-97.97	±	3.90	0.50
Inner Ear CL: Side effect	P-X	P	8.06	±	4.39	X	10.78	±	8.34	-2.72	±	4.57	-15.87	±	25.01	0.25
Inner Ear CL: Side effect	C-X	C	6.86	±	4.89	X	10.78	±	8.34	-3.92	±	3.60	-34.91	±	8.14	0.01*
Inner Ear IL: Hearing loss	P-X	P	5.25	±	10.60	X	8.46	±	13.78	-3.21	±	4.85	-54.67	±	69.69	0.02*
Inner Ear IL: Hearing loss	C-X	C	5.12	±	10.26	X	8.46	±	13.78	-3.34	±	5.03	-72.76	±	37.66	0.01*
Inner Ear IL: Side effect	P-X	P	18.20	±	9.16	X	23.69	±	7.03	-5.50	±	4.45	-25.24	±	22.81	0.02*
Inner Ear IL: Side effect	C-X	C	15.74	±	8.61	X	23.69	±	7.03	-7.95	±	3.93	-36.14	±	19.37	0.01*
Lacrimal gland CL: Ocular side effect	P-X	P	1.52	±	1.35	X	13.90	±	24.65	-12.39	±	23.62	-36.83	±	67.23	0.05
Lacrimal gland CL: Ocular side effect	C-X	C	0.69	±	0.15	X	13.90	±	24.65	-13.22	±	24.52	-56.46	±	32.24	0.00*
Lacrimal gland IL: Ocular side effect	P-X	P	34.99	±	40.82	X	48.07	±	38.81	-13.08	±	20.78	-46.87	±	43.57	0.05
Lacrimal gland IL: Ocular side effect	C-X	C	38.71	±	46.25	X	48.07	±	38.81	-9.36	±	19.98	-46.12	±	49.18	0.20
Lens CL: Cataract	P-X	P	0.02	±	0.01	X	0.08	±	0.08	-0.06	±	0.08	-70.56	±	20.28	0.00*
Lens CL: Cataract	C-X	C	0.02	±	0.01	X	0.08	±	0.08	-0.06	±	0.07	-72.91	±	17.98	0.00*
Lens IL: Cataract	P-X	P	9.40	±	27.81	X	15.11	±	33.79	-5.71	±	12.09	-63.96	±	28.62	0.00*
Lens IL: Cataract	C-X	C	1.91	±	5.71	X	15.11	±	33.79	-13.19	±	28.49	-86.21	±	7.86	0.00*
Optic nerve CL: Blindness	P-X	P	0.27	±	0.47	X	0.36	±	0.61	-0.09	±	0.29	67.64	±	132.28	1.00
Optic nerve CL: Blindness	C-X	C	0.25	±	0.39	X	0.36	±	0.61	-0.11	±	0.25	-10.81	±	65.27	0.73
Optic nerve IL: Blindness	P-X	P	2.04	±	2.62	X	2.41	±	2.87	-0.37	±	0.49	-17.48	±	34.05	0.02*
Optic nerve IL: Blindness	C-X	C	2.29	±	2.64	X	2.41	±	2.87	-0.12	±	0.33	3.56	±	20.95	0.50
Parotis CL: Xerostomia	P-X	P	1.74	±	3.07	X	1.63	±	1.92	0.11	±	1.16	-18.96	±	26.83	0.13
Parotis CL: Xerostomia	C-X	C	1.15	±	1.30	X	1.63	±	1.92	-0.48	±	0.63	-26.98	±	9.29	0.00*
Parotis IL: Xerostomia	P-X	P	2.51	±	4.61	X	2.11	±	2.74	0.41	±	2.35	6.67	±	37.25	0.65
Parotis IL: Xerostomia	C-X	C	2.99	±	5.44	X	2.11	±	2.74	0.88	±	3.01	20.41	±	50.07	0.65
Pituitary: Endocrine Dysfunction	P-X	P	2.63	±	1.84	X	3.97	±	2.59	-1.35	±	2.65	-16.98	±	61.67	0.20
Pituitary: Endocrine Dysfunction	C-X	C	3.43	±	1.81	X	3.97	±	2.59	-0.55	±	1.83	0.94	±	53.32	0.57
Skin: Alopecia	P-X	P	44.61	±	22.64	X	46.94	±	19.81	-2.33	±	3.75	-6.95	±	8.04	0.10
Skin: Alopecia	C-X	C	43.18	±	24.06	X	46.94	±	19.81	-3.77	±	6.47	-11.11	±	15.54	0.10

Skin: Erythema	P-X	P	26.54	±	17.13	X	24.81	±	13.95	1.73	±	3.56	4.47	±	8.72	0.25
Skin: Erythema	C-X	C	26.49	±	15.92	X	24.81	±	13.95	1.68	±	3.20	5.55	±	12.91	0.25

Supplementary Table S5. Risk ratio (RR) for secondary central nervous system (CNS) malignancies between proton (P) and photon (X) plans, as well as carbon ion (C) and photon (X) plans.

SD: standard deviation; *: p-value < 0.05; Reported values are rounded to two decimal places.

Risk Ratio					
	Comparison	Mean	±	SD	p-value
Brain: Secondary malignancy	(Photon/Proton)	1.72	±	0.62	0.00*
Brain: Secondary malignancy	(Photon/Carbon ion)	2.68	±	0.69	0.00*

Supplementary Table S6. Overview of selected publications reporting outcomes of radiotherapy for meningiomas.

Reported parameters include: number of patients (n), median age at initiation of radiotherapy (RT), tumor location, treatment modality, prescribed median dose, proportion of patients with previous surgery, median follow-up since RT (in months), local control (LC) rates, and treatment-related adverse events.

SBM: skull base meningioma; FSRT: fractionated stereotactic radiotherapy; IMRT: intensity modulated radiotherapy; 3D-CRT: 3D conformal radiotherapy.

Publication	n	Median age at RT (years)	Tumor location	Treatment modality + median dose	Previous surgery	Median follow-up since RT (months)	Local control (LC)	Adverse events
Debus, J. et al. (2001) [14]	189	53<br>(10-83)	SBM:<br><br>WHO1: 43%<br>WHO2: 5%<br>benign: 21%<br>NA: 31%	Photons:<br>FSRT<br><br>56.8 Gy<br>(52.4-61.2 Gy)	Neuro-surgical intervention: 69%<br><br>no surgical intervention: 31%	35<br>(3-144)	Last Follow-Up:<br><br>benign: 98% (177/180)<br><br>WHO2: 78% (7/9)	Clinically significant late adverse events (grade 3): 2.1%<br><br>onset of new adverse events : grade 1-3:<br>• reduced vision: 3.2%<br>• loss of vision fields: 0.5%<br>• diplopia: 0.5%<br>• trigeminal hyp/dysesthesia: 0.5%<br>• trigeminal neuropathy: 3.2%<br>• permanent hearing loss: 3.7%<br>• pituitary dysfunction: 0.5%<br><br>impairment of pre-existing neurologic symptoms: 7%
Solda, F. et al. (2013) [15]	222	52<br>(27-77)	Meningioma: WHO1<br><br>skull base: 65%<br><br>optic nerve sheath: 19%<br><br>suprasellar/ sellar: 9%<br><br>falx/para-sagittal: 6%<br><br>pineal region: 1%	Photons:<br>FSRT<br><br>59%:<br>50 Gy<br><br>41%:<br>55 Gy	Neuro-surgical intervention: 53.6%<br><br>no surgical intervention: 46.4%	43<br>(3-144)	All:<br>5-years: 93%<br><br>10-years: 86%	Acute adverse events :<br>• transient localized alopecia: 100%<br>• transient worsening of headache: 4%<br>• transient mild visual deterioration: 1%<br><br>Chronic adverse events :<br>• worsening of pre-existing cranial nerve deficit: 3.5%<br>• new trigeminal neuralgia: 0.5%<br>• mild neurocognitive dysfunction: 1%<br>• cerebrovascular accident: 1%<br>• radiation retinopathy: 1% (optic nerve sheath meningioma)
Combs, S.E. et al. (2018) [16]	927	59<br>(14-86)	SBM WHO1 (low-grade)	Photons:<br><br>radio-surgery:<br>13%: 13 Gy<br><br>fractionated high-precision RT:<br>87%: 54 Gy	NA	81<br>(1-348)	5-years: 92%<br>10-years: 86%<br><br>no difference between radiosurgery and fractionated high-precision RT	Below 5% in both groups without any severe treatment-related complications:<br><br>• no treatment-related side effects > CTCAE grade 2<br>• cranial nerve impairment: < 2%
Rogers, L. et al. (2018) [17]<br><br>RTOG 0539	52	/	Meningioma:<br><br>WHO2 + GTR: 69%<br><br>recurrent WHO1: 31%	Photons:<br><br>IMRT<br>or<br>3D-CRT<br><br>54 Gy	Neuro-surgical intervention: 100%	44<br>(4.8-58.8)	3-years: 93.8%	No reported CTCAE grade 3 adverse events<br><br>Acute adverse events:<br>• grade 1: 21.7%<br>• grade 2: 10.9%<br><br>Late adverse events:<br>• grade 1: 13.7%: most common:<br>• dizziness<br>• memory impairment<br>• peripheral sensory neuropathy

								• peripheral motor neuropathy<br>• grade 2: 25.5%: most common:<br>• seizure<br>• speech disorder<br>• depression<br>• trigeminal nerve disorder<br>• olfactory nerve disorder<br>• peripheral sensory neuropathy<br>• memory impairment<br>• dizziness
El Shafie, R.A. et al. (2018) [18]	110	52 (45-59)	SBM:<br><br>WHO1: 54.5%<br><br>WHO2: 6.4%<br><br>WHO3: 0.9%<br><br>NA: 38.2%<br><br>location: sphenoid wing: 38.2%	Protons: 54 Gy (RBE) (50-60 Gy (RBE)) (low-risk: 94.5%)<br><br>Photons: 50 Gy (48.4-55.8 Gy) + carbon ion boost: 18 Gy (RBE) (high-risk: 5.5%)	Neuro-surgical intervention: 62.7%<br><br>no surgical intervention: 29.1%<br><br>unknown: 8.2%	46.8 (95% CI: 39.9-53.7)	5-years: 96.6%	No grade 4 or 5 adverse events according to CTCAE v4.0<br><br>most common adverse events:<br><br>Acute:<br>• grade 1-2:<br>• focal alopecia: 63.6%<br>• fatigue: 47.3%<br>• skin irritation: 40.0%<br>• headaches: 22.7%<br>• ≥ grade 3:<br>• nausea: 0.9%<br>• mucositis: 0.9%<br><br>Late:<br>• grade 1-2:<br>• fatigue: 9.1%<br>• headache: 9.1%<br>• dysgeusia: 3.6%<br>• xerostomia: 3.6%<br>• ≥ grade 3:<br>• fatigue: 0.9%<br>• radionecrosis: 2.7%
Holtzman, A.L. et al. (2023) [19]	59	54 (22-85)	Meningioma: radiographic benign or pathologically proven WHO1	Protons: 50.4 Gy (RBE) (48.6-61.2 Gy (RBE))	Neuro-surgical intervention: 53%<br><br>primary RT, no surgical intervention: 47%	Clinical: 6.3 years (0.7-15.5)<br><br>imaging: 4.6 years (0.2-13.8)	5-years: 94%	2 patients with CTCAE (version 5.0) grade 3 or greater adverse events:<br><br>• optic neuropathy<br>• secondary meningioma
Krcek, R. et al. (2023) [20]	200	50.4 (3.2-79.8)	Meningioma: WHO1: 70% WHO2: 27.5% WHO3: 2.5%<br><br>location: SBM: 70%<br><br>non-SBM: 30%	Protons:<br><br>Benign: WHO1: 54 Gy (RBE) (50.4-64 Gy (RBE))<br><br>non-benign: WHO 2/3: 60 Gy (RBE) (54-68 Gy (RBE))	Neuro-surgical intervention:<br><br>Simpson 1-3: 15%<br><br>Simpson 4/5: 66%<br><br>primary RT, no surgical intervention: 19%	65 (3.8-260.8)	5-year LC:<br><br>WHO1: 97.5%<br><br>WHO2/3: 77.8%	Acute adverse events:<br>• grade 1: 62.5%<br>• grade 2: 25.5%<br>• grade 3: 1%<br>no grade ≥ 4<br><br>Acute adverse events:<br>• alopecia: 54.5%<br>• dermatitis: 50.5%<br>• fatigue: 29.5%<br>• headache: 22.5%<br>• nausea: 14%<br><br>Late adverse events:<br>• grade 1: 17%<br>• grade 2: 25.5%<br>• ≥ grade 3: 12%: most common late adverse events ≥ grade 3:<br>• visual adverse events: 5%<br>• cataract: 2%<br>• brain necrosis: 1.5%<br><br>EORTC-QLQ-C30 and BN20: stable QoL
Deng, M.Y. et al. (2024) [21]<br><br>MARCIE	33	53 (29-75)	Meningioma: WHO2<br><br>various intracranial locations (51.5%: periventricular)	Bimodal radiotherapy: Photons: 50 Gy + carbon ion boost: 18 Gy (RBE)	Neuro-surgical intervention: 100% (subtotal resection)	42 (5-109)	3-years: 86.7%	Acute adverse events:<br>• headache: 15%<br>• dizziness: 9%<br>• nausea: 15%<br>• seizures: 3%<br>• fatigue: 24%<br>=> no acute adverse events > CTCAE grade 3<br><br>Late adverse events:

								• headache: 3%<br>• dizziness: 6%<br>• motoric deficits: 12%<br>• sensory deficits: 27%<br>• seizures: 12%<br>• fatigue: 15%<br>• RICE/radiation necrosis: 45% (6% > CTCAE grade 3)

References

1. Bentzen SM, Constine LS, Deasy JO, Eisbruch A, Jackson A, Marks LB, et al. Quantitative Analyses of Normal Tissue Effects in the Clinic (QUANTEC): an introduction to the scientific issues. Int J Radiat Oncol Biol Phys 2010;76:S3-9. https://doi.org/10.1016/j.ijrobp.2009.09.040.
2. Emami B, Lyman J, Brown A, Coia L, Goitein M, Munzenrider JE, et al. Tolerance of normal tissue to therapeutic irradiation. Int J Radiat Oncol Biol Phys 1991;21:109-22. https://doi.org/10.1016/0360-3016(91)90171-y.
3. Moteabbed M, Yock TI, Paganetti H. The risk of radiation-induced second cancers in the high to medium dose region: a comparison between passive and scanned proton therapy, IMRT and VMAT for pediatric patients with brain tumors. Phys Med Biol 2014;59:2883-99. https://doi.org/10.1088/0031-9155/59/12/2883.
4. Schneider U, Kaser-Hotz B. Radiation risk estimates after radiotherapy: application of the organ equivalent dose concept to plateau dose-response relationships. Radiat Environ Biophys 2005;44:235-9. https://doi.org/10.1007/s00411-005-0016-1.
5. Niyazi M, Niemierko A, Paganetti H, Sohn M, Schapira E, Goldberg S, et al. Volumetric and actuarial analysis of brain necrosis in proton therapy using a novel mixture cure model. Radiother Oncol 2020;142:154-61. https://doi.org/10.1016/j.radonc.2019.09.008.
6. Bender ET. Brain necrosis after fractionated radiation therapy: is the halftime for repair longer than we thought? Med Phys 2012;39:7055-61. https://doi.org/10.1118/1.4762562.
7. Burman C, Kutcher GJ, Emami B, Goitein M. Fitting of normal tissue tolerance data to an analytic function. Int J Radiat Oncol Biol Phys 1991;21:123-35. https://doi.org/10.1016/0360-3016(91)90172-z.
8. Peuker L, Rolf D, Oertel M, Peuker A, Scobioala S, Hering D, et al. Definition of an Normal Tissue Complication Probability Model for the Inner Ear in Definitive Radiochemotherapy of Nasopharynx Carcinoma. Cancers 2022;14:https://doi.org/10.3390/cancers14143422.
9. De Marzi L, Feuvret L, Boule T, Habrand JL, Martin F, Calugaru V, et al. Use of gEUD for predicting ear and pituitary gland damage following proton and photon radiation therapy. Br J Radiol 2015;88:20140413. https://doi.org/10.1259/bjr.20140413.
10. Gondi V, Hermann BP, Mehta MP, Tome WA. Hippocampal dosimetry predicts neurocognitive function impairment after fractionated stereotactic radiotherapy for benign or low-grade adult brain tumors. Int J Radiat Oncol Biol Phys 2013;85:348-54. https://doi.org/10.1016/j.ijrobp.2012.11.031.
11. Batth SS, Sreeraman R, Dienes E, Beckett LA, Daly ME, Cui J, et al. Clinical-dosimetric relationship between lacrimal gland dose and ocular toxicity after intensity-modulated radiotherapy for sinonasal tumours. Br J Radiol 2013;86:20130459. https://doi.org/10.1259/bjr.20130459.
12. Houweling AC, Philippens ME, Dijkema T, Roesink JM, Terhaard CH, Schilstra C, et al. A comparison of dose-response models for the parotid gland in a large group of head-and-neck cancer patients. Int J Radiat Oncol Biol Phys 2010;76:1259-65. https://doi.org/10.1016/j.ijrobp.2009.07.1685.
13. Dutz A, Luhr A, Agolli L, Troost EGC, Krause M, Baumann M, et al. Development and validation of NTCP models for acute side-effects resulting from proton beam therapy of brain tumours. Radiother Oncol 2019;130:164-71. https://doi.org/10.1016/j.radonc.2018.06.036.
14. Debus J, Wuendrich M, Pirzkall A, Hoess A, Schlegel W, Zuna I, et al. High efficacy of fractionated stereotactic radiotherapy of large base-of-skull meningiomas: long-term results. J Clin Oncol 2001;19:3547-53. https://doi.org/10.1200/JCO.2001.19.15.3547.

15. Solda F, Wharram B, De Ieso PB, Bonner J, Ashley S, Brada M. Long-term efficacy of fractionated radiotherapy for benign meningiomas. Radiother Oncol 2013;109:330-4. https://doi.org/10.1016/j.radonc.2013.10.006.
16. Combs SE, Farzin M, Boehmer J, Oehlke O, Molls M, Debus J, et al. Clinical outcome after high-precision radiotherapy for skull base meningiomas: Pooled data from three large German centers for radiation oncology. Radiother Oncol 2018;127:274-9. https://doi.org/10.1016/j.radonc.2018.03.006.
17. Rogers L, Zhang P, Vogelbaum MA, Perry A, Ashby LS, Modi JM, et al. Intermediate-risk meningioma: initial outcomes from NRG Oncology RTOG 0539. J Neurosurg 2018;129:35-47. https://doi.org/10.3171/2016.11.JNS161170.
18. El Shafie RA, Czech M, Kessel KA, Habermehl D, Weber D, Rieken S, et al. Clinical outcome after particle therapy for meningiomas of the skull base: toxicity and local control in patients treated with active rasterscanning. Radiat Oncol 2018;13:54. https://doi.org/10.1186/s13014-018-1002-5.
19. Holtzman AL, Glassman GE, Dagan R, Rao D, Fiester PJ, Tavanaieour D, et al. Long-term outcomes of fractionated proton beam therapy for benign or radiographic intracranial meningioma. J Neurooncol 2023;161:481-9. https://doi.org/10.1007/s11060-022-04207-0.
20. Krcek R, Leiser D, Garcia-Marqueta M, Bolsi A, Weber DC. Long Term Outcome and Quality of Life of Intracranial Meningioma Patients Treated with Pencil Beam Scanning Proton Therapy. Cancers 2023;15:https://doi.org/10.3390/cancers15123099.
21. Deng MY, Maas SLN, Hinz F, Karger CP, Sievers P, Eichkorn T, et al. Efficacy and toxicity of bimodal radiotherapy in WHO grade 2 meningiomas following subtotal resection with carbon ion boost: Prospective phase 2 MARCIE trial. Neuro Oncol 2024;26:701-12. https://doi.org/10.1093/neuonc/noad244.
